# IGFBP2/ITGA5 promotes gefitinib resistance via activating STAT3/CXCL1 axis in non-small cell lung cancer

**DOI:** 10.1038/s41419-024-06843-y

**Published:** 2024-06-25

**Authors:** Hengxiao Lu, Jiangshan Ai, Yingying Zheng, Wolong Zhou, Liming Zhang, Jiebo Zhu, Heng Zhang, Shaoqiang Wang

**Affiliations:** 1 Department of Thoracic Surgery, Weifang People’s Hospital, Shandong Second Medical University, Weifang, 261041 Shandong Province China; 2 Department of Thoracic Surgery, Affiliated Hospital of Qingdao University, Qingdao, 266003 Shandong Province China; 3 Health Management Center, Weifang People’s Hospital, Shandong Second Medical University, Weifang, 261041 Shandong Province China; 4 Department of Thoracic Surgery, Xiangya Hospital, Central South University, 87# Xiangya Road, Changsha, 410008 Hunan Province China; 5 Department of Thoracic Surgery, Affiliated Hospital of Jining Medical University, Jining Medical University, Jining, 272029 Shandong Province China; 6 Xiangya Lung Cancer Center, Xiangya Hospital, Central South University, 87# Xiangya Road, Changsha, 410008 Hunan Province China; 7 National Clinical Research Center for Geriatric Disorders (Xiangya Hospital), Changsha, 410008 Hunan Province China; 8 Department of Scientific Research Management, Weifang People’s Hospital, Shandong Second Medical University, Weifang, 261041 Shandong Province China

**Keywords:** Cancer, Diseases

## Abstract

There is a paucity of comprehensive knowledge pertaining to the underlying mechanisms leading to gefitinib resistance in individuals diagnosed NSCLC harboring EGFR-sensitive mutations who inevitably develop resistance to gefitinib treatment within six months to one year. In our preceding investigations, we have noted a marked upregulation of IGFBP2 in the neoplastic tissues of NSCLC, predominantly in the periphery of the tissue, implying its plausible significance in NSCLC. Consequently, in the current research, we delved into the matter and ascertained the molecular mechanisms that underlie the participation of IGFBP2 in the emergence of gefitinib resistance in NSCLC cells. Firstly, the expression of IGFBP2 in the bronchoalveolar lavage fluid and lung cancer tissues of 20 NSCLC patients with gefitinib tolerance was found to be significantly higher than that of non-tolerant patients. Furthermore, in vitro and in vivo experiments demonstrated that IGFBP2 plays a significant role in the acquisition of gefitinib resistance. Mechanistically, IGFBP2 can activate STAT3 to enhance the transcriptional activity of CXCL1, thereby increasing the intracellular expression level of CXCL1, which contributes to the survival of lung cancer cells in the gefitinib environment. Additionally, we identified ITGA5 as a key player in IGFBP2-mediated gefitinib resistance, but it does not function as a membrane receptor in the process of linking IGFBP2 to intracellular signaling transduction. In conclusion, this study demonstrates the promoting role and mechanism of IGFBP2 in acquired gefitinib resistance caused by non-EGFR secondary mutations, suggesting the potential of IGFBP2 as a biomarker for gefitinib resistance and a potential intervention target.

## Introduction

Lung cancer remains the leading cause of cancer death globally, and non-small cell lung cancer (NSCLC) accounts for ~85% of lung cancers [1, 2]. Despite the advances of surgical techniques, chemo-, radio- and molecular targeted therapies, the prognosis of NSCLC remains unfavorable [3]. Gefitinib, an epidermal growth factor receptor tyrosine kinase inhibitor (EGFR-TKI), exhibits robust clinical efficacy in NSCLC patients with EGFR-sensitive mutations [4, 5]. Unfortunately, patients develop resistance to gefitinib remains an unsolved fundamental challenge [6]. Research has indicated that patients who experience disease progression on gefitinib exhibit both “on-target” mechanisms, which are primarily caused by acquired resistance mutations in the kinase domains of EGFR or anaplastic lymphoma kinase (ALK), and “off-target” mechanisms of resistance, which are mediated by non-target kinase alterations such as bypass signaling activation or phenotypic transformation [7]. Unraveling the mechanism behind the resistance to gefitinib could help overcome this resistance and enhance the effectiveness of gefitinib therapy in NSCLC.

Insulin-like growth factor binding protein 2 (IGFBP2) belongs to IGFBP family which has attracted increasing attention for the critical roles in various cancers [8, 9]. IGFBP2 is a secreted protein which prevents IGF-I/-II binding to their receptors, and it is also implicated in the regulation of tumor microenvironment (TME) in a macrophage-dependent manner [10, 11]. More importantly, IGFBP2 is highly expressed in lung tumors and blood of patients with lung cancer, and the high level of IGFBP2 is associated with unfavorable survival and metastasis of lung cancer patients [12, 13]. Additionally, serum IGFBP2 level is recognized as a diagnostic biomarker of severe malnutrition in patients with advanced lung cancer. Previous studies have illustrated that IGFBP2 overexpression facilitates camptothecin resistance via regulating caspase-3 in an IGF-independent manner [14], and it also promotes EGFR-TKI erlotinib resistance by activating IGF-1R in lung cancer cells [15]. However, the role of IGFBP2 in gefitinib resistance remains uninvestigated.

CXC motif chemokine ligand 1 (CXCL1) is a cytokine belonging to the CXC chemokine subfamily, known to induce migration and infiltration of neutrophils to sites of high expression [16]. In addition to its functions in the immune system, CXCL1 has also been implicated in regulating tumor growth and metastasis [17]. A study in breast cancer revealed the involvement of a paracrine network centered around chemokines CXCL1/2 in promoting lung metastasis and chemotherapy resistance, and blocking this regulatory network using CXCL1/2 receptor inhibitors in combination with chemotherapy significantly reduced cancer burden in preclinical mouse models, addressing a key issue of chemotherapy failure and recurrence [18]. In lung cancer, only CXCL1 among the CXC chemokine subfamily was found to be significantly differentially expressed in the blood of patients with localized or systemic recurrence [19]. Moreover, it has been discovered that transforming growth factor-β1 (TGFβ1) can promote the stem-like characteristics of lung cancer by inducing the expression of CXCL1 [20]. These studies collectively suggest that CXCL1 may be associated with the malignant progression of advanced lung cancer, and the underlying mechanisms warrant further investigation.

Our previous study has shown that IGFBP2 is expressed in microvesicles released from NSCLC cells and is highly expressed in the marginal region of NSCLC tumors, indicating its potential role in remodeling the tumor microenvironment [13]. The objective of this study was to investigate whether the secretion of IGFBP2 promotes resistance to the drug gefitinib in NSCLC and to explore the underlying mechanism. Our findings demonstrated a positive correlation between the expression and secretion of IGFBP2 and gefitinib resistance in NSCLC patients. Through in vitro and in vivo experiments, we demonstrated that the upregulation or downregulation of IGFBP2 significantly increased or decreased gefitinib resistance in lung cancer cells, accompanied by the induction of the epithelial-mesenchymal transition (EMT) in these cells. Mechanistically, IGFBP2 stimulated the transcriptional activity of STAT3 on CXCL1, leading to an upregulation of CXCL1 expression in lung cancer cells and subsequently promoting gefitinib resistance. It is important to note that the IGFBP2-mediated resistance to gefitinib depends on the involvement of integrin-α5 (ITGA5) on the cell membrane, despite the absence of a direct binding between IGFBP2 and ITGA5. These findings unveil a novel regulatory axis involving IGFBP2 and CXCL1 in the development of gefitinib resistance, providing valuable insights for the development of prognostic models and intervention strategies in precision therapy for lung cancer patients resistant to gefitinib.

## Materials and methods

### Clinical specimens

The BLF (*n* = 20) and blood samples (*n* = 20) were collected from NSCLC patients in Xiangya hospital of Central South University from 2020 to 2022. Clinical pathological section from pathology department of Xiangya hospital of Central South University were employed in this study. Consents were obtained from all participants. The study has been approved by the Ethics Committee of the Xiangya Hospital of Central South University and Medical Research Ethics Committee of Affiliated Hospital of Jining Medical College.

### ELISA assay

The IGFBP2 levels in BLF and serum were measured using Human IGFBP2 ELISA kit (EHIGFBP2, Invitrogen, Carlsbad, CA, USA) according to the manufacturer’s protocol. A450 was measured using a microplate reader (Bio-Rad, Hercules, CA, USA).

### Immunohistochemistry (IHC) analysis

The TMA or lung tissue sections were deparaffinized and exposed to antigen retrieval. After blocking, the slides were incubated with anti-IGFBP2 (1:200, ab188200, Abcam, Cambridge, UK), anti-STAT3 (1:200, ab68153, Abcam) or anti-CXCL1 (1:100, PA5-86508, Invitrogen) antibody at 4 °C overnight. This is followed by the incubation with HRP-conjugated secondary antibody. Signals were visualized using DAB color development kit (Beyotime, Haimen, China).

### Cell culture, treatment, transfection and establishment of gefitinib-resistant cell lines

Human NSCLC cancer cell lines NCI-H1650 (del ex19) and NCI-H1975 (L858R / T790M) cells with accurate STR identification were obtained from ATCC (Manassas, VA, USA), and cultured in RPMI160 containing 10% FBS (Gibco, Grand Island, NY, USA). To establish gefitinib-resistant cell lines, NCI-H1650 and NCI-H1975 cells were cultured in medium containing 10 nM gefitinib [21] (Selleckchem, Radnor, PA, USA) for 20 weeks, resulting in the acquisition of resistant cell lines with resistance index of 3.64 and 3.91, respectively, designated as NCI-H1650/Gef and NCI-H1975/Gef. The resistant cell lines were cultured in gefitinib-free medium for 1 week to allow for recovery, followed by the subsequent analyses.

All cells were maintained at 37 °C/5% CO_2_. For human recombinant IGFBP2 (hr IGFBP2, ab63223, Abcam) treatment, NSCLC cells were treated with 1 μg/mL hr IGFBP2 for 24 h. sh-IGFBP2, si-CXCL1, sh-STAT3, and si-ITGA5 were purchased from General BIOL. The cDNA of IGFBP2 or CXCL1 was cloned into pcDNA3.1 vector. The siRNA/shRNA/overexpression construct were transfected into NSCLC cells using Lipofectamine 3000 (Invitrogen).

### CCK-8 assay

Cell viability was assessed using Cell Counting Kit-8 (Beyotime). Briefly, cells were plated into 96-well plates. At 24 h post-treatment, 10 μL CCK-8 solution was added into each well, and incubated for 1 h. A450 was measured using a microplate reader (Bio-Rad).

### Colony formation assay

NCI-H1650 and NCI-H1975 cells were transfected with siRNA or overexpression plasmid for 48 h. Cells were then replated into 60-mm plates and treated with 1 μg/ml hr IGFBP2 or 1 μM gefitinib for 24 h. After 2 weeks, cells were fixed and stained with crystal violet. The colonies were photographed and counted under a microscope (Nikon, Tokyo, Japan).

### Animal study

Male BALB/c nude mice (6~8-week-old, *n* = 6 per group) were from Hunan Silaikejingda Experimental Animal Co., Ltd. (Changsha, China). The animal protocols were approved by Xiangya Hospital of Central South University. Briefly, lung metastases were induced by injecting stable transfected NCI-H1650 and NCI-H1975 cells (1 × 10^6^ cells/0.2 mL RPMI1640) intravenously. After inoculation, 50 mg/kg gefitinib was administrated orally. On day 21, mice were sacrificed and subjected to subsequent analysis. For the inhibition study, mice were randomly divided into 4 groups: Sham, gefitinib, gefitinib+sh-IGFBP2 and gefitinib+TTI-101 groups. 20 mg/kg gefitinib was administrated by gavage for 10 days after inoculation, followed by the injection of adenoviral sh-IGFBP2 (10 μL) or STAT3 inhibitor TTI-101 (10 mg, Sigma-Aldrich, St Louis, MO, USA) for 20 days. For bioluminescent imaging, luciferase stable expressed NSCLC cells were injected intravenously. Mice were injected with 200 mg/kg D-luciferin (Invitrogen) at 15 min post-inoculation. Bioluminescent imaging was conducted using In Vivo Imaging System (Xenogen, Alameda, CA, USA) as described [22].

### Transwell assay

NCI-H1650 and NCI-H1975 cells (1 × 10^6^ cells/mL) were plated onto Matrigel-coated 24-well cell culture inserts (Corning, Corning, NY, USA) and grown in serum-free medium. The lower wells were filled with complete RPMI1640. After 24 h, cells were fixed and stained with 1% crystal violet, and images were photographed using a microscope (Nikon).

### Wound healing assay

Cells were cultured in 6-well plates until they reached 90% confluence. A scratch was created using the pipette tip, and the detached cells were removed by rinsing with PBS. At 0 and 24 h, the scratch wounds were photographed using a microscope (Nikon).

### qRT-PCR

Total RNA was isolated using Trizol (Invitrogen), and cDNA was synthesized using PrimeScript RT Kit (TaKaRa, Dalian, China). qRT-PCR was conducted using SYBR Premix Ex Taq (TaKaRa), and calculated using 2^–ΔΔCT^ method. Primers are listed in Table 1.

**Table 1:** RT-qPCR primers.

IGFBP2	Forward: 5′-CCTGCCAGGACTCCCTGC-3′
	Reverse: 5′-ATGCTTGTCACAGTTGGGGATG-3′
STAT3	Forward: 5′-TGCCGGAGAAACAGGATGG-3′
	Reverse: 5′-CTCTCAATCCAAGGGGCCAG-3′
ITGA5	Forward: 5′-GGGCTTCAACTTAGACGCGG-3′
	Reverse: 5′-GAGGTAGACAGCACCACCCT-3′
CXCL1	Forward: 5′-CCAAGAACATCCAAAGTGTGAAC-3′
	Reverse: 5′-AGGATTGAGGCAAGCTTTCC-3′
GAPDH	Forward: 5′-CCAGGTGGTCTCCTCTGA-3′
	Reverse: 5′-GCTGTAGCCAAATCGTTGT-3′

### Western blot

Protein was extracted using RIPA buffer (Beyotime). After protein quantification, proteins were separated by gel electrophoresis and transferred onto NC membrane (Bio-Rad). The blots were blocked with 5% non-fat milk, and probed with primary antibody at 4°C overnight. This is followed by the incubation with secondary antibody, and the bands were visualized using ECL kit (Beyotime). Primary antibodies used in western blot: anti-IGFBP2 (1:1000, ab188200), anti-N-cadherin (1:2000, ab76011), anti-vimentin (1:2000, ab92547), anti-CXCL1 (1:1000, ab86436), anti-STAT3 (1:1000, ab171361), anti-p-STAT3 (1:1000, ab76315), anti-VEGFR (1:1000, ab39638) and anti-His tag (1:1000, ab18184) antibodies from Abcam, as well as anti-ITGA5 (1:1000, 10569-1-AP) antibody from Proteintech (Rosemont, IL, USA).

### Chromatin immunoprecipitation (ChIP) assay

ChIP assay was conducted using Pierce ChIP Assay Kit (26157, Pierce, Rockford, IL, USA). NSCLC cells were crosslinked with 1% formaldehyde, lysed and digested with MNase. The chromatin fractions were incubated with normal IgG or anti-STAT3 (2 μg, ab171361, Abcam), anti-H3K27ac (2 μg, ab4729, Abcam) or anti-RNA Pol II (2 μg, ab5131, Abcam) antibody at 4 °C overnight. The DNA-protein complexes were enriched by Protein A/G beads, and purified DNAs were analyzed by PCR.

### Dual luciferase reporter assay

The wild-type or mutated STAT3 promoter region (STAT3-WT or STAT3-MUT) was cloned into pGL-3 vector (Promega, Madison, WI, USA). Cells were co-transfected with STAT3-WT or STAT3-MUT luciferase construct and IGFBP2 overexpression plasmid. Luciferase activities were measured using Dual luciferase reporter system (Promega) at 48 h post-transfection.

### Yeast two hybrid assay

The interaction between IGFBP2 and ITGA5 was validated using Yeast two hybrid assay as described [23]. Briefly, the pAS-2-IGFBP2 construct was transformed into *Saccharomyces cerevisiae* strain AH109. The transformed cells were then transformed with pAS-2-ITGA5 and cultured in selective media for 5~7 days.

### Co-immunoprecipitation (Co-IP)

Proteins were extracted from tissues and cell using IP lysis buffer (Beyotime). Protein lysates (1 mg) were incubated with anti-IGFBP2 antibody (2 μg, ab188200, Abcam) or normal IgG at 4 °C overnight. The protein complexes were enriched by Protein A/G beads (Pierce). The eluted proteins were analyzed by western blot. Normal IgG and whole protein lysates were used as a negative and input control, respectively.

### GST pull-down assay

GST pull-down assay was conducted using Pierce GST Pull-Down Kit (21516, Pierce). Briefly, GST-tagged IGFBP2 was immobilized to the resin, and incubated with His-tagged ITGA5 at 4 °C overnight. The protein complex was then eluted and analyzed by western blot.

### Statistical analysis

Results are presented as mean ± SD unless mentioned otherwise. Comparisons were analyzed by One-way ANOVA or Student’s *t*-test using SPSS 22.0 software. Significance in all figures was indicated as follows: *P < 0.05, **P < 0.01, ***P < 0.001

## Results

### IGFBP2 expression is positively correlated with gefitinib resistance in NSCLC

**Fig. 1:**
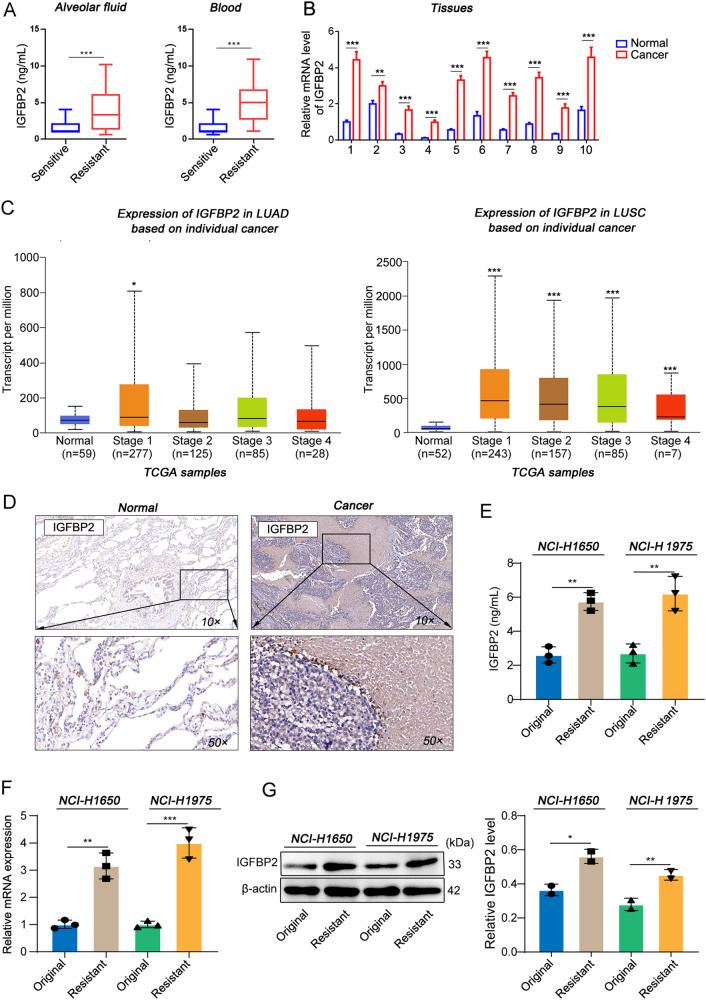
IGFBP2 expression is positively correlated with gefitinib resistance in NSCLC. The BLF (*n* = 20) and blood samples (*n* = 20) were collected from NSCLC patients. **A** The IGFBP2 level in BLF and serum was assessed by ELISA assay. **B** The mRNA level of IGFBP2 in lung tissues was detected by qRT-PCR. **C** The expression of IGFBP2 in NSCLC tissues based on TCGA database. **D** The immunoreactivity of IGFBP2 in lung tissues was detected by IHC analysis. **E** The secretion of IGFBP2 in culture medium was detected by ELISA assay. **F**, **G** The mRNA and protein levels of IGFBP2 were detected by qRT-PCR and western blot, respectively. In **A**, **B**, **E**, **F,** **G**, Student’s *t*-test. In **C**, Dunnett’s test of one-way ANOVA. *P < 0.05; **P < 0.01; ***P < 0.005. BLF bronchoalveolar lavage fluid, TCGA The Cancer Genome Atlas.

To delineate the role of IGFBP2 in gefitinib resistance, we first examined the IGFBP2 level in BLF and serum of NSCLC patients. As shown in Fig. 1A, the secretion of IGFBP2 of gefitinib-resistant patients was much higher than that from patients who benefited from gefitinib treatment. Consistent with The Cancer Genome Atlas (TCGA) data, IGFBP2 was also significantly upregulated in cancer tissues, compared with corresponding paracancerous tissues as detected by qRT-PCR and IHC assay (Fig. 1B–D). Subsequently, we selected six different lung cancer cell lines and performed genetic sequencing of the EGFR exons 19–21. As previously known, the NCI-H1650 cell line exhibited a heterozygous E746-A750 deletion mutation in exon 19, while the HCC827 cell line had a homozygous E746-A750 deletion mutation. The NCI-H1975 cell line had a T790M mutation in exon 20 and an L858R mutation in exon 21. Other cell lines, such as CALU-1, 95D, and A549, did not have any missense mutations in the EGFR exons 19–21, but had mutations in the intronic regions. For instance, the 95D and A549 cell lines had an Aå G mutation in intron 21, and the CALU-1 cell line had two SNPs in intron 19 (Fig. S1A). Therefore, we performed sensitivity testing for gefitinib on these cell lines. The results showed that the three lung cancer cell lines with wild-type EGFR exons 19–21 (A549, 95D, CALU-1) and the NCI-H1975 cell line with the T790M/L858R double mutation exhibited relative resistance to gefitinib, with IC50 values of 20.80 nM, 13.51 nM, 26.36 nM, and 14.26 nM, respectively. On the other hand, the NCI-H1650 and NCI-HCC827 cell lines with the E746-A750 deletion mutation showed relative sensitivity to gefitinib, with IC50 values of 11.16 nM and 2.39 nM, respectively (Fig. S1B). Furthermore, we conducted detection of IGFBP2 protein levels in these 6 lung cancer cell lines. Our findings revealed that IGFBP2 expression was detected in NCI-1650, NCI-H1975, and 95D cells, while it was not detected in A549, CALU-1, and HCC827 cells (Fig. S1C). Therefore, we selected NCI-H1650 and NCI-H1975 cell lines, which had EGFR exon mutations and approximate IC50 values, to induce acquired resistance to gefitinib. After obtaining the two resistant cell lines, NCI-H1650/Gef and NCI-H1975/Gef (with RI of 4.87 and 2.31, respectively) (Fig. S1D), we performed targeted sequencing of the genomic fragments between EGFR exons 19–21 and found no new mutations that differed from the parental cell lines (Fig. S1A). This suggests that the acquired resistance to gefitinib in these cell lines was not associated with additional EGFR mutations and may involve other bypass activation mechanisms.

Further investigations revealed that NCI-H1650/Gef and NCI-H1975/Gef cells exhibited higher secretion levels of IGFBP2 compared to their parental cells (Fig. 1E). Additionally, mRNA and protein levels of IGFBP2 were significantly elevated in NCI-H1650/Gef and NCI-H1975/Gef cells (Fig. 1F, G), suggesting a potential role of IGFBP2 in enhancing gefitinib resistance.

### IGFBP2 promotes malignant phenotype of NSCLC cells

To test the effects of IGFBP2 on malignant progression of NSCLC, the gain- and loss-of function experiments were conducted in NCI-H1650 and NCI-H1975 cell lines. The protein level of IGFBP2 was successfully downregulated or upregulated by siRNA (si-IGFBP2) or overexpression plasmid (OE-IGFBP2) transfection, respectively (Fig. S2A). Transwell invasion and wound healing assays revealed that overexpression of IGFBP2 facilitated cell invasion and migration, while silencing of IGFBP2 impaired the invasive and migratory capacities of NCI-H1650 and NCI-H1975 cells (Fig. S2B, C). Additionally, IGFBP2 overexpression exerted a positive effect on cell proliferation, whereas IGFBP2 knockdown inhibited colony formation in both NSCLC cells (Fig. S2D). Western blot revealed that overexpression of IGFBP2 increased the expression of mesenchymal markers N-cadherin and vimentin, while lack of IGFBP2 decreased the levels of these proteins in both NSCLC cells (Fig. S2E). These data suggest that IGFBP2 promotes cell invasion, migration, proliferation and epithelial-mesenchymal transition (EMT) in NCI-H1650 and NCI-H1975 cells.

### IGFBP2 enhances gefitinib resistance in NSCLC

**Fig. 2:**
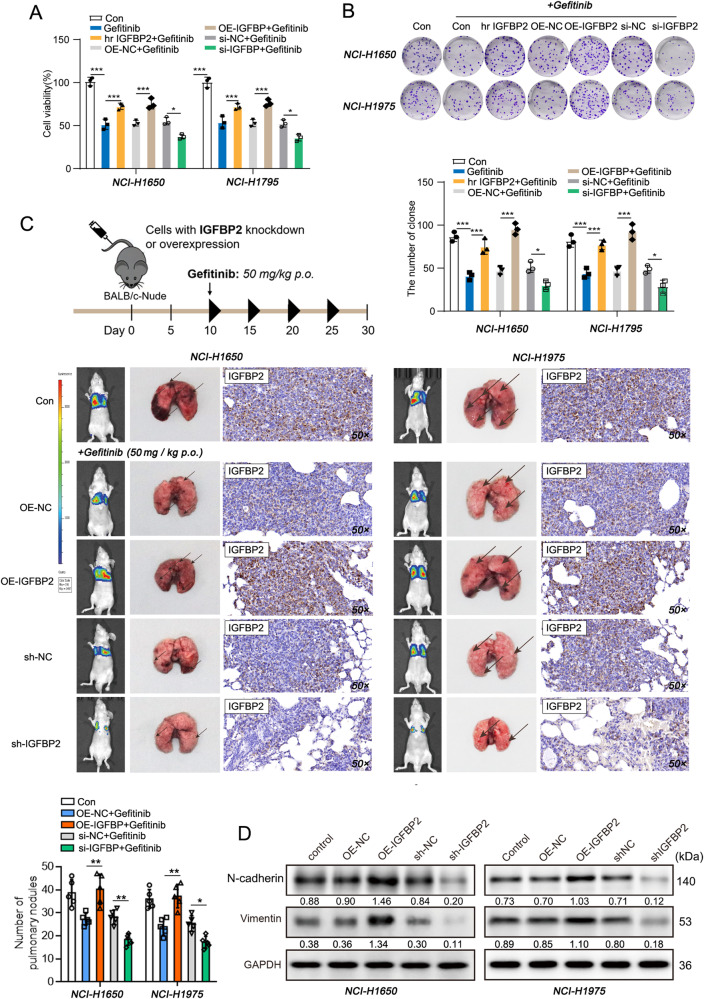
IGFBP2 enhances gefitinib resistance in NSCLC. NCI-H1650 and NCI-H1975 cells were either treated with 1 μg/mL human recombinant IGFBP2 (hr IGFBP2) or transfected with OE-IGFBP2/si-IGFBP2, followed by the treatment of gefitinib (10 nM). **A** Cell viability was monitored by CCK-8 assay. **B** Colony formation was detected by colony formation assay. **C** The scheme of the in vivo experiment design. Representative photos and in vivo imaging of xenograft tumors/lung metastases with quantitative analysis. The immunoreactivity of IGFBP2 in lung tissues was detected by IHC analysis. **D** The protein levels of N-cadherin and vimentin were detected by western blot. Dunnett’s test of one-way ANOVA. *P < 0.05; **P < 0.01; ***P < 0.001. hr human recombinant, OE-NC overexpression vector alone, si-NC negative control siRNA.

The sensitivity to gefitinib was examined in IGFBP2-overexpssing or -knockdown NSCLC cells. NCI-H1650 and NCI-H1975 cells were either treated with human recombinant IGFBP2 (hr IGFBP2) or transfected with OE-IGFBP2/si-IGFBP2, followed by the treatment of gefitinib. CCK-8 and colony formation assays showed that cell viability and colony forming capacity of NSCLC cells were decreased upon gefitinib treatment, while hr IGFBP2 or OE-IGFBP2 exhibited a rescue effect. By contrast, knockdown of IGFBP2 exacerbated the cytotoxicity of gefitinib (Fig. 2A, B). We next tested the function of IGFBP2 in mice model. Mice were injected with stable transfected NSCLC cells, and gefitinib was administered orally. As shown in Fig. 2C, IGFBP2 overexpression impaired the cytotoxicity of gefitinib in which gefitinib-suppressed metastasis was reversed by IGFBP2 overexpression, whereas IGFBP2 knockdown exerted opposite effects on the number of lung metastases. IHC analysis confirmed the successful overexpression and knockdown of IGFBP2 in lung metastases (Fig. 2C), and this is accompanied by the upregulation and downregulation of mesenchymal markers, respectively (Fig. 2D). Collectively, these findings indicate that IGFBP2 enhances gefitinib resistance in NSCLC cells and lung metastasis model.

**Fig. 3:**
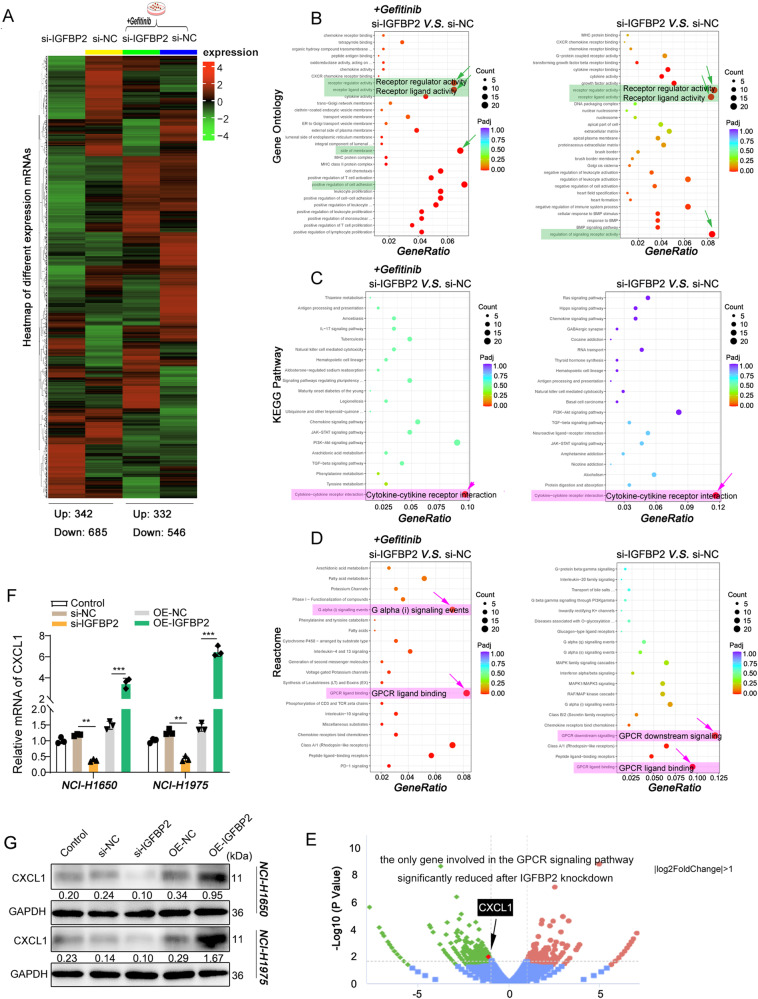
IGFBP2 positively regulates CXCL1 expression in NSCLC cells. Heatmap (**A**), GO (**B**) and KEGG enrichment pathway (**C**) and Reactome analysis (**D**) for RNA-seq. **E** Changes of GPCR signaling molecules were detected by sequencing. NCI-H1650 and NCI-H1975 cells were transfected with si- IGFBP2 or IGFBP2 overexpression construct. **F**, **G** The mRNA and protein levels of CXCL1 were detected by qRT-PCR and western blot, respectively. Dunnett’s test of one-way ANOVA. **P < 0.01; ***P < 0.001. OE-NC overexpression vector alone, si-NC negative control siRNA.

### IGFBP2 positively regulates CXCL1 expression in NSCLC cells

As a secreted protein that potentially participates in cell signaling, our aim was to identify and screen for gene expression in lung cancer cells that depend on IGFBP2. RNA-seq results revealed that silencing IGFBP2 induced the expression of 342 genes and reduced the expression of 685 genes. Additionally, under the influence of gefitinib, silencing IGFBP2 upregulated the expression of 332 genes and downregulated the expression of 546 genes (Fig. 3A). GO and KEGG enrichment analysis showed that the downregulated genes after IGFBP2 silencing were mainly involved in EMT-related ligand/receptor activation pathways, cell adhesion pathways, and signaling pathways related to chemokines/chemokine receptors. Furthermore, Reactome analysis suggested that the downregulated genes mainly functioned in G protein-coupled receptor (GPCR) signaling pathways (Fig. 3B–D). Given CXCL1 was identified as the only gene implicated in GPCR signaling which was downregulated by IGFBP2 silencing regardless of the presence or absence of gefitinib (Fig. 3E), we focused on its relationship with IGFBP2 in subsequent studies. Consistent with the RNA-seq results, qRT-PCR and western blot analyses demonstrated that knocking out IGFBP2 reduced the mRNA and protein expression levels of CXCL1 in NCI-H1650 and NCI-H1975 cells. Conversely, upregulating the expression of IGFBP2 significantly increased the transcription and expression of CXCL1 (Fig. 3F, G). These findings support the role of IGFBP2 as a positive regulator of CXCL1 expression.

### IGFBP2 promotes gefitinib resistance in NSCLC by upregulating CXCL1

**Fig. 4:**
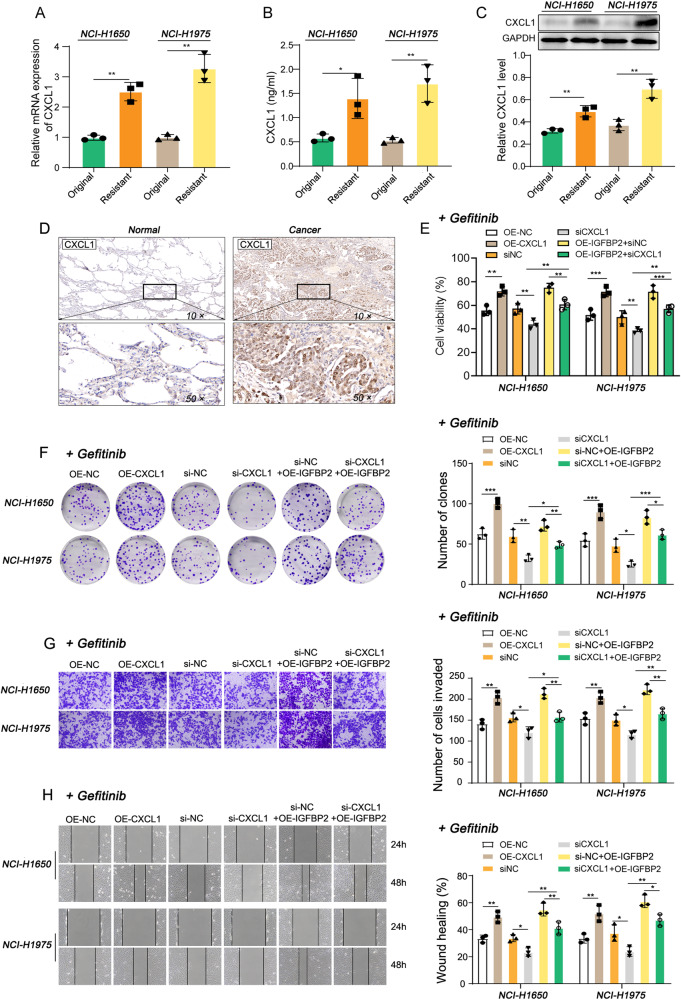
IGFBP2/CXCL1 facilitates gefitinib resistance in NSCLC. **A**, **C** The mRNA and protein levels of CXCL1 were detected by qRT-PCR and western blot, respectively. **B** The secretion of CXCL1 was detected by ELISA assay. **D** The immunoreactivity of CXCL1 in lung tissues was detected by IHC analysis. NCI-H1650 and NCI-H1975 cells were transfected with CXCL1 overexpression construct, si-CXCL1 or/and IGFBP2 overexpression construct. **E** Cell proliferation was monitored by CCK-8 assay. **F** Colony forming ability was assessed by colony formation assay with quantitative analysis. **G**, **H** Cell invasion and migration were detected by Transwell and wound healing assays with quantitative analysis, respectively. In **A**–**C**, Student’s *t*-test. In **E**–**H**, Dunnett’s test of one-way ANOVA. *P < 0.05; **P < 0.01; ***P < 0.001. OE-NC, overexpression vector alone; si-NC, negative control siRNA.

To investigate if CXCL1 was implicated in IGFBP2-regulated gefitinib resistance, CXCL1 expression was next detected in NCI-H1650/Gef and NCI-H1975/Gef cells. Compared with the parental cells, the mRNA and protein levels of CXCL1, as well as the secretion of CXCL1, were dramatically elevated in NCI-H1650/Gef and NCI-H1975/Gef cells (Fig. 4A–C). IHC analysis further showed that the CXCL1 was highly expressed in lung tumors, compared with their normal counterparts (Fig. 4D). Functional experiments illustrated that overexpression of CXCL1 promoted cell proliferation, invasive and migratory capacities, while knockdown of CXCL1 exhibited opposite effects (Figs. S3 and 4E–H). In particular, IGFBP2-enhanced cell proliferation, invasion and migration were abolished by CXCL1 knockdown (Fig. 4E–H). These findings indicate that IGFBP2 induces and relies on the upregulation of CXCL1 expression to promote gefitinib resistance in non-small cell lung cancer.

### STAT3 acts as a transcriptional activator of CXCL1

**Fig. 5:**
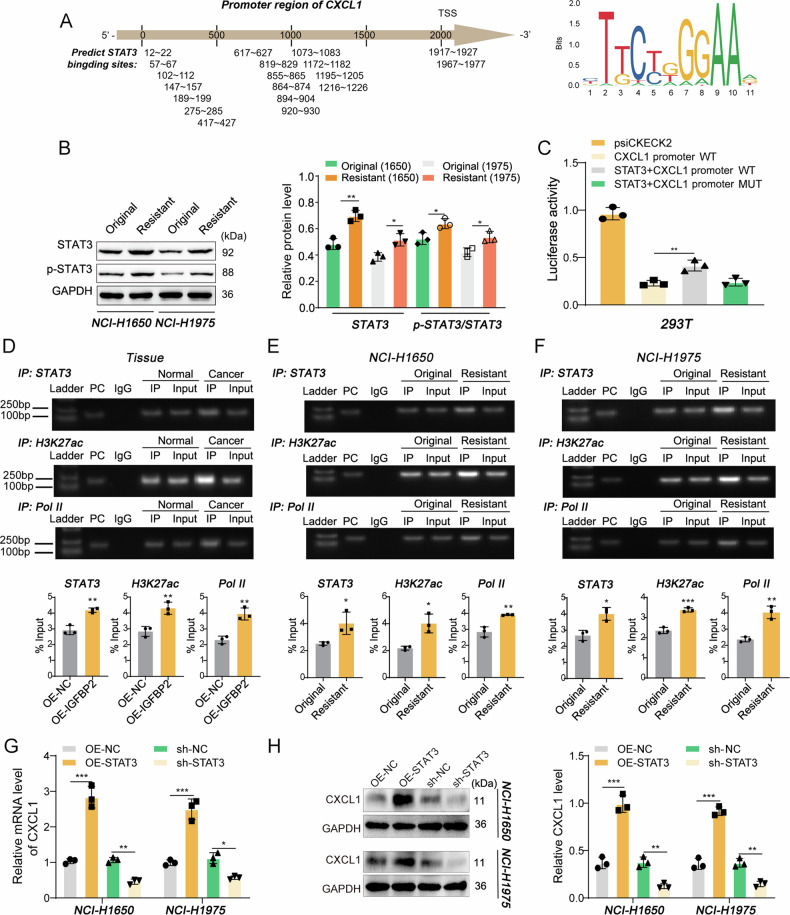
STAT3 acts as a transcriptional activator of CXCL1. **A** The putative binding sites between STAT3 and CXCL1 promoter were predicted by JASPAR, a database of transcription factor binding profiles (genereg.net). **B** The protein levels of STAT3 and p-STAT3 were detected by western blot with quantitative analysis. **C** The luciferase activity was assessed using dual luciferase reporter assay. (D-F) The interactions among STAT3, H3K27Ac, RNA pol II and CXCL1 promoter in tumor tissues and NSCLC cells were detected by ChIP assay. **G**, **H** The mRNA and protein levels of CXCL1 were detected by qRT-PCR and western blot, respectively. In **B**, **C**, **G**, **H** Dunnett’s test of one-way ANOVA. In **D**–**F**, Student’s *t*-test. *P < 0.05, **P < 0.01, ***P < 0.001. IgG, normal IgG control; Input, total chromatin, IP immunoprecipitation; MUT mutant; OE-NC overexpression vector alone; sh-NC negative control shRNA; WT wild type.

Given that IGFBP2 is not a transcription factor, the other key molecule might be implicated in IGFBP2-mediated regulation of CXCL1. Interestingly, analysis based on JASPAR, a database of transcription factor binding profiles, predicted a number of putative binding sites between STAT3 and CXCL1 promoter (Fig. 5A), indicating that STAT3 might regulate CXCL1 expression transcriptionally. Western blot showed significant upregulation of p-STAT3/STAT3 in NCI-H1650/Gef and NCI-H1975/Gef cells, suggesting a potential role of phosphorylated activated STAT3 in gefitinib resistance in NSCLC cells (Fig. 5B). Dual luciferase assay showed that co-transfection of STAT3 overexpression construct and CXCL1 promoter WT resulted in a dramatical induction of luciferase activity, whereas this effect was abrogated by CXCL1 promoter MUT (Fig. 5C). Enhanced interactions between STAT3/H3K27ac/RNA pol II and CXCL1 promoter were found in NSCLC tumors compared with the matched adjacent normal tissues by ChIP assay (Fig. 5D). Consistently, these associations were also increased in NCI-H1650/Gef and NCI-H1975/Gef cells, in comparison with their parental cells (Fig. 5E, F). These data indicated that the binding between STAT3 and CXCL1 promoter correlated with the transcriptional activity of CXCL1 in lung cancer tissues and gefitinib-resistant cells. By altering the total STAT3 level, a synchronized change of CXCL1 expression in NSCLC cell lines was observed in NCI-H1650 and NCI-H1675 (Figs. S4 and 5G, H), indicating that STAT3 responds to IGFBP2 and functions as a transcriptional activator of CXCL1 in NSCLC cells.

### IGFBP2 positively regulates CXCL1 expression via STAT3-mediated transcriptional activation

**Fig. 6:**
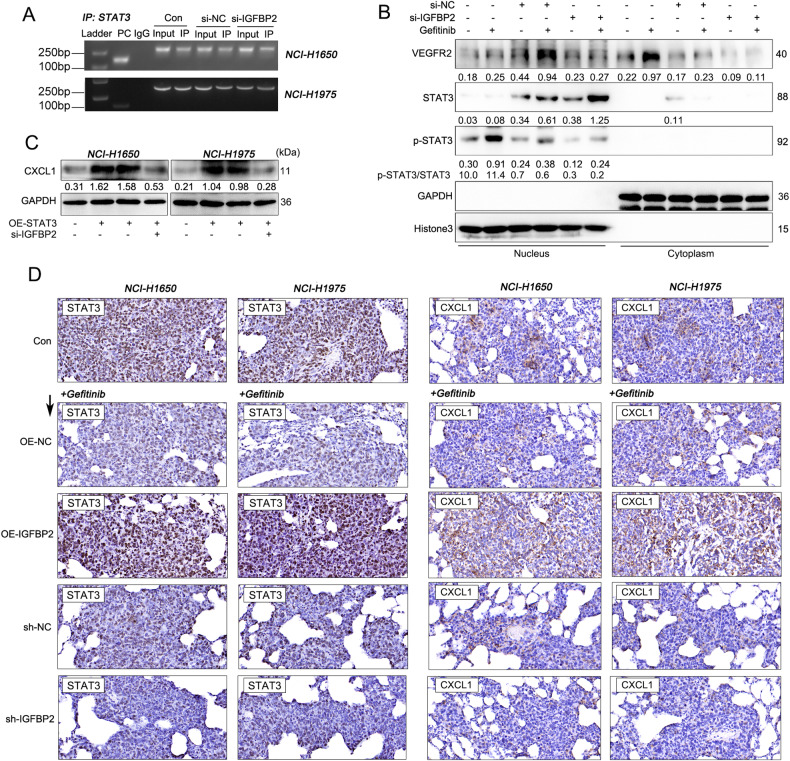
IGFBP2 positively regulates CXCL1 expression via STAT3-mediated transcriptional activation. **A** The association between STAT3 and CXCL1 promoter was detected by ChIP assay. **B** The protein levels of VEGFR, STAT3 and p-STAT3 in the nucleus and cytoplasm were detected by western blot. **C** The protein level of CXCL1 was detected by western blot. **D** The immunoreactivities of STAT3 and CXCL1 in lung metastases were detected by IHC analysis.

Previous study has demonstrated that STAT3 signaling acts as a downstream signaling of IGFBP2 [24]. We thus sought to study whether IGFBP2 regulated CXCL1 via STAT3 signaling in NSCLC cells. ChIP assay showed that IGFBP2 knockdown significantly downregulated the abundance of STAT3 binding to the CXCL1 promoter in NSCLC cells (Fig. 6A). Notably, IGFBP2 knockdown did not downregulate total STAT3 protein level, but significantly reduced p-STAT3/STAT3 in the nucleus (Fig. 6B). Moreover, gefitinib exhibited similar effects with IGFBP2 on total STAT3 and p-STAT3/STAT3. In addition, a previous report revealed that VEGFR, a membrane protein widely recognized as a VEGF receptor, may facilitate the translocation of STAT3 into the nucleus to mediate transcription. We thus examined its expression in the cytoplasm and nucleus. Gefitinib induced the expression of VEGFR in the nucleus, while IGFBP2 knockdown exerted an opposite effect (Fig. 6B). In addition, overexpression of STAT3 partially attenuated IGFBP2 knockdown-mediated downregulation of CXCL1 (Figs. S5 and 6C). Furthermore, the same tendency was observed in lung metastases formed by lung cancer cells with different IGFBP2 levels (Fig. 6D). Collectively, these results suggest that IGFBP2 knockdown decreases the level of active STAT3 in the nucleus, thereby modulating CXCL1 expression.

### The role of ITGA5 in mediating the connection between IGFBP2 and STAT3/CXCL1 transcriptional regulatory axis, independent of direct interaction with IGFBP2

**Fig. 7:**
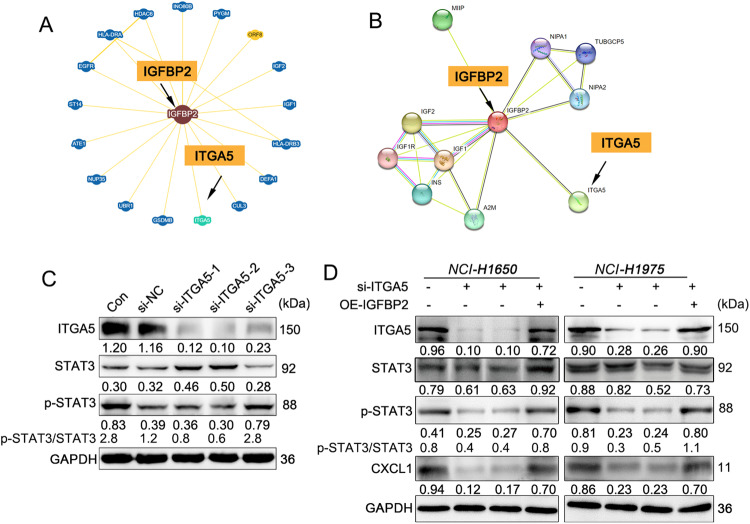
IGFBP2 activates the STAT3/CXCL1 signal axis through ITGA5. The interaction relation between IGFBP2 and ITGA5 through **A** BioGRID and **B** String databases. **C**, **D** The protein levels of ITGA5, STAT3, p-STAT3 and CXCL1 were detected by western blot.

**Fig. 8:**
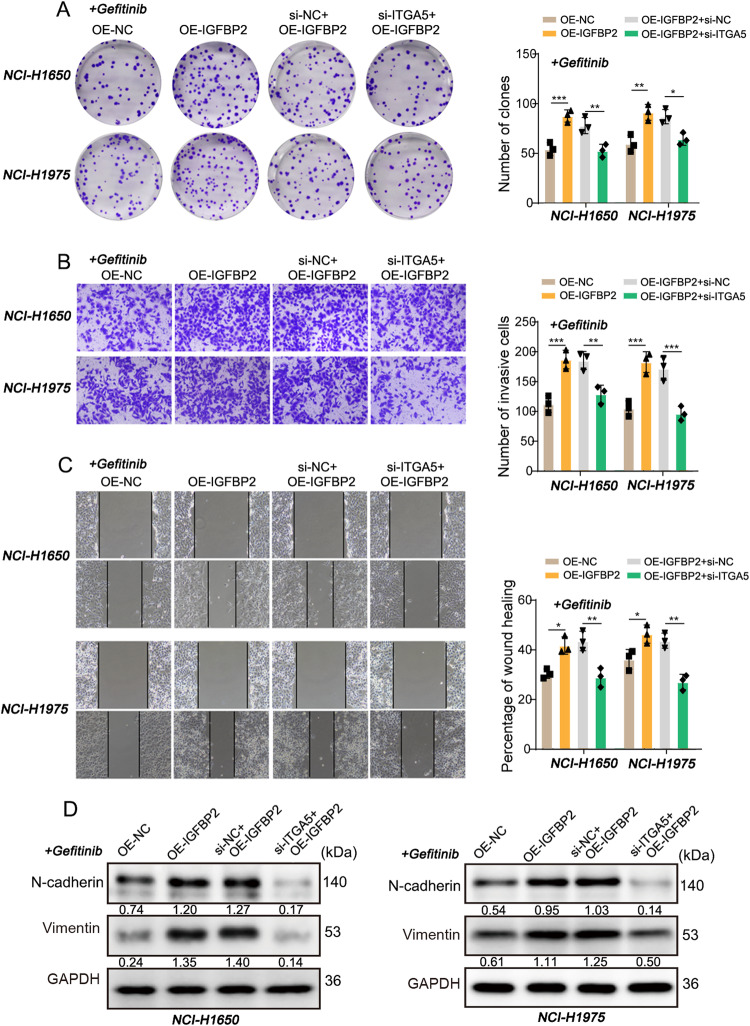
IGFBP2/ITGA5 axis promotes gefitinib resistance in NSCLC cells. NCI-H1650 or NCI-H1975 cells were transfected with IGFBP2 overexpression construct or/and si-ITGA5. **A** Colony formation was detected by colony formation assay. **B**, **C** Cell invasion and migration were measured by Transwell and wound healing assays with quantitative analysis, respectively. **D** The protein levels of N-cadherin and vimentin were detected by western blot. Dunnett’s test of one-way ANOVA. *P < 0.05; **P < 0.01; ***P < 0.001. OE-NC overexpression vector alone, si-NC negative control siRNA.

In the following study, our attempt is to identify the membrane interacting proteins of IGFBP2 in order to elucidate how secreted IGFBP2 activates the STAT3/CXCL1 signaling axis within cells. Among the potential interacting proteins with IGFBP2 obtained from the BioGRID and String databases, we focused on an integrin α subfamily member, ITGA5, which is known to play a crucial role in various cancers according to recent studies. However, little is known about their expression and function in NSCLC (Fig. 7A, B). The detection results showed that silencing of ITGA5 successfully reduced the protein level of ITGA5 and significantly decreased p-STAT3/STAT3 in cells, indicating the potential role of ITGA5 in the STAT3 signaling pathway (Fig. 7C). In addition, qRT-PCR, western blot and IF unequivocally revealed that IGFBP2 positively regulated ITGA5 in NSCLC cells (Fig. S6). Furthermore, overexpression of IGFBP2 counteracted ITGA5 knockdown-mediated STAT3 inactivation and CXCL1 reduction, as well as ITGA5 knockdown-suppressed cell proliferation and metastasis (Figs. 7D, 8A–D and S7). These data indicate that an ITGA5-dependent regulatory mechanism mediates the crosstalk between IGFBP2 and STAT3/CXCL1. Subsequently, we proceeded to silence the expression of IGFBP2, ITGA5, STAT3, and CXCL1 genes in H1650/Gef and NCI-H1975/Gef cells. The results demonstrated that knocking down these genes all contributed to the inhibition of proliferation, invasion, and metastatic capabilities of resistant cells (Fig. S8), suggesting their involvement in a shared signaling pathway that promotes gefitinib resistance.

**Fig. 9:**
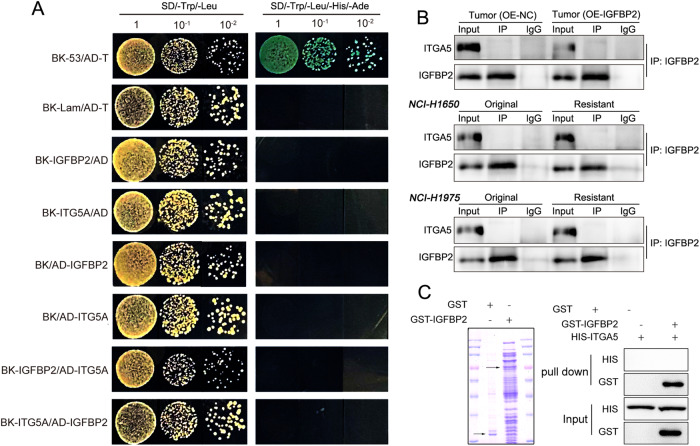
IGFBP2 does not directly bind to ITGA5 in NSCLC cells. **A** The putative interaction between IGFBP2 and ITGA5 was validated by yeast two hybrid assay. **B** The interaction between IGFBP2 and ITGA5 in lung metastases and NSCLC cells was detected by co-IP. Whole cell lysates or normal IgG served as an input or negative control, respectively. **C** The in vitro binding of GST-tagged IGFBP2 and His-tagged ITGA5 was assessed by GST pull-down assay.

We next sought to test the direct interaction between IGFBP2 and ITGA5 in NSCLC cells. Unfortunately, both yeast two-hybrid system and CoIP experiments targeting IGFBP2 yielded negative results (Fig. 9A, B). Consistently, GST-tagged IGFBP2 was unable to pull down His-tagged ITGA5 in vitro (Fig. 9C). These data do not support the direct interaction between IGFBP2 and ITGA5 in NSCLC, although the alteration in either IGFBP2 or ITGA5 has significant effects on STAT3/CXCL1. The role of ITGA5 in the crosstalk between IGFBP2 and STAT3/CXCL1 merits further investigation in the future study.

### IGFBP2 enhances gefitinib resistance via STAT3/CXCL1 axis in vivo

**Fig. 10:**
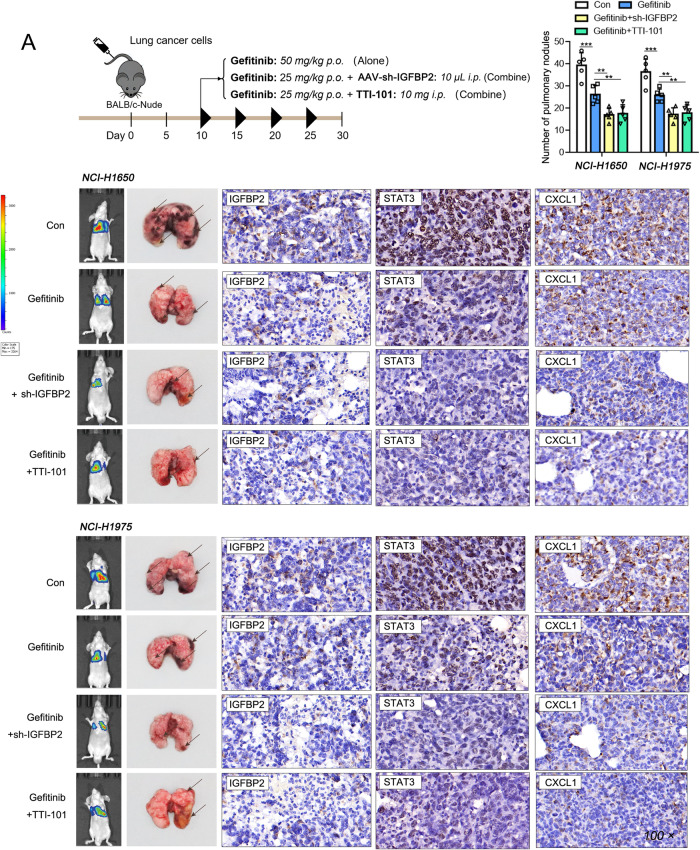
IGFBP2 enhances gefitinib resistance via STAT3/CXCL1 axis in vivo. **A** The scheme of the in vivo experiment design. Representative photos of lung metastases with quantitative analysis and in vivo imaging of lung tissues. The immunoreactivities of IGFBP2, STAT3 and CXCL1 in lung metastases were detected by IHC analysis. Dunnett’s test of one-way ANOVA. *P < 0.05; **P < 0.01; ***P < 0.001.

To further validate the role of IGFBP2/STAT3/CXCL1 axis in vivo, xenograft model was established. As presented in Fig. 10A middle panel, the number of lung metastases was dramatically decreased upon gefitinib treatment, while sh-IGFBP2-gefitinib or STAT3 inhibitor TTI-101-gefitinib half-dose combination potentiated the effect of gefitinib on lung metastasis. In line with this finding, gefitinib-reduced luciferase signals in lung tissues were potentiated by sh-IGFBP2 or TTI-101 as detected by in vivo imaging (Fig. 10A, left panel). IHC analysis revealed that the immunoreactivities of IGFBP2, STAT3 and CXCL1 in lung metastases were downregulated by gefitinib, whereas these positive effects of gefitinib were also potentiated by sh-IGFBP2 or TTI-101 (Fig. 10A, right panel). Taken together, these data indicate that IGFBP2 facilitates gefitinib resistance via STAT3/CXCL1 axis in vivo.

## Discussion

**Fig. 11:**
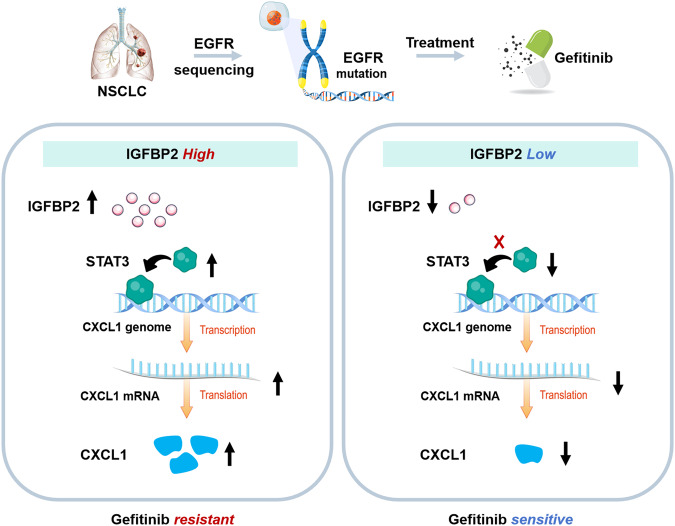
Graphic abstract. IGFBP2/ITGA5 promoted gefitinib resistance via activating STAT3/CXCL1 axis.

Since the discovery that NSCLC is driven by activating mutations in EGFR, EGFR tyrosine kinase inhibitors (EGFR-TKIs), such as gefitinib and erlotinib, have been effectively utilized in clinical treatment [25]. However, patients eventually develop resistance. The acquired resistance to EGFR-TKIs is inevitable due to various factors, including secondary mutations, activation of alternative pathways, aberrant downstream signaling, compromised EGFR-TKIs-mediated apoptosis pathway, histological transformation, and ATP-binding cassette transporter effusion [26]. In the current study, we reported that IGFBP2 contributed to gefitinib resistance in NCI-H1650 (del ex19) and NCI-H1975 (L858R/T790M) cells, and ITGA5 was required in this process. In terms of mechanism, IGFBP2/ITGA5 enhances the expression of CXCL1 by activating STAT3, thereby promoting gefitinib resistance in non-small cell lung cancer (Fig. 11).

In lung cancer, high expression of IGFBP2 is associated with poor survival and metastasis of the patients [12, 13]. Consistent with previous reports, we found that the circulating level and local expression of IGFBP2 were positively correlated with gefitinib resistance in NSCLC, while no correlation between IGFBP2 level and TNM stage of lung cancer was found based on TCGA database. In addition, we observed an elevated expression of IGFBP2 in NCI-H1650/Gef and NCI-H1975/Gef resistant cells that harbor the same EGFR mutation as their parental strains. This suggests that IGFBP2 mediates gefitinib resistance through activation of alternative pathways independent of EGFR mutations. Further investigation revealed a significant impact of IGFBP2 on the transcriptional activity of STAT3-mediated CXCL1. Therefore, our findings support the potential of IGFBP2 as a biomarker for gefitinib resistance in NSCLC. It is noteworthy that circulating IGFBP2 as a prognostic marker offers advantages such as convenient collection and easy detection.

Previous studies have demonstrated that IGFBP2 binds to integrin α5β1 and αvβ3 to trigger PI3K and MAPK signalings in glioblastoma cells [27–31]. In particular, a direct interaction between IGFBP2 and ITGA5 has been observed in glioma cells, and this association is required for IGFBP2-regualted cell mobility [29]. By contrast, the binding between IGFBP2 and ITGA5 was not detected in NSCLC tissues and cells by yeast two hybrid assay, co-IP and GST pull-down assay. Given IGFBP2 is a secreted protein, the interaction in extracellular matrix is hardly detected by co-IP. Whether the secreted IGFBP2 bound to ITGA5 merits in-depth investigation in the future study.

Several studies have reported the roles of integrins in ERGR-TKI resistance in NSCLC [32, 33]. For instance, integrin β1 contributes to gefitinib resistance via PI3K/Akt signaling in NSCLC, and integrin β3 enhances erlotinib resistance in NSCLC through AXL/YAP pathway [32, 33]. In the present study, we demonstrated for the first time that ITGA5 promoted gefitinib resistance in NSCLC via STAT3/CXCL1 axis in an EGFR-independent manner, broadening the understanding of gefitinib resistance in NSCLC.

In glioma and melanoma, IGFBP2 facilitates the nuclear accumulation of EGFR, thereby activating STAT3 [24, 34]. Apart from the well-studied EGFR-STAT3 signaling, we identified a novel regulatory axis, namely STAT3/CXCL1, in IGFBP2-enhanced gefitinib resistance in NSCLC. IGFBP2 silencing counteracted gefitinib-increased VEGFR expression in NSCLC cells, thus inhibiting the nuclear translocation of activated STAT3, suggesting that IGFBP2 contributes to gefitinib resistance via VEGFR/STAT3 signaling. It is well-accepted that VEGFR/STAT3 signaling is implicated in angiogenesis in a variety of cancers [35]. It is of interest to investigate the crosstalk between angiogenesis and IGFBP2-regulated gefitinib resistance in the future study. IGFBP2 enhanced the association between STAT3 and CXCL1 promoter, and positively increased CXCL1 expression at the transcriptional level.

CXCL1 expression was negatively correlated with lung cancer survival in patients [20]. Like VEGF, CXCL1 exhibits increased expression in response to EGF stimulation [36]. A study in hepatocellular carcinoma reported that gefitinib significantly inhibits the upregulation of VEGF and CXCL1 induced by EGF stimulation, thereby suppressing angiogenesis [37]. In this study, our results indicate that the expression level of CXCL1 is up-expressed in gefitinib-resistant NSCLC cells compared to the sensitive cells, and knockdown of CXCL1 significantly enhances the sensitivity of NSCLC cells to gefitinib. This finding is consistent with a recent report that demonstrated the upregulation of CXCL1 in lung cancer that is resistant to gefitinib [38]. To date, CXCL1 has been identified as a poor prognostic biomarker in NSCLC, and this might be attributed, at least in part, to its role in drug resistance.

In summary, our study reveals that the IGFBP2/ITGA5 signaling pathway plays a crucial role in promoting resistance to gefitinib in NSCLC through the activation of STAT3/CXCL1 axis. Mechanistically, the activation of IGFBP2/ITGA5 leads to the rapid activation of STAT3, which in turn binds to the promoter region of CXCL1, resulting in the enhancement of gefitinib resistance. Targeting the IGFBP2/STAT3/CXCL1 axis simultaneously may potentially enhance the efficacy of gefitinib in NSCLC treatment. Moreover, our findings suggest that IGFBP2-mediated chemoresistance may not be limited to gefitinib alone. Further research would be required to determine whether this pathway also contributes to resistance to other EGFR-TKIs, such as erlotinib or osimertinib.

### Supplementary information

Supplement file

Original Data

aj-checklist

## Data Availability

The datasets generated during and/or analyzed during the current study are available from the corresponding author on reasonable request.
