# Supplementary material for: IGFBP2/ITGA5 promotes gefitinib resistance via activating STAT3/CXCL1 axis in non-small cell lung cancer
Source: Cell Death Dis. 2024 Jun 25;15(6):447. doi: 10.1038/s41419-024-06843-y (PMC11199710; doi:10.1038/s41419-024-06843-y)
Supplement: Supplementary file 1 — Supplement file [file 41419_2024_6843_MOESM1_ESM.pdf]

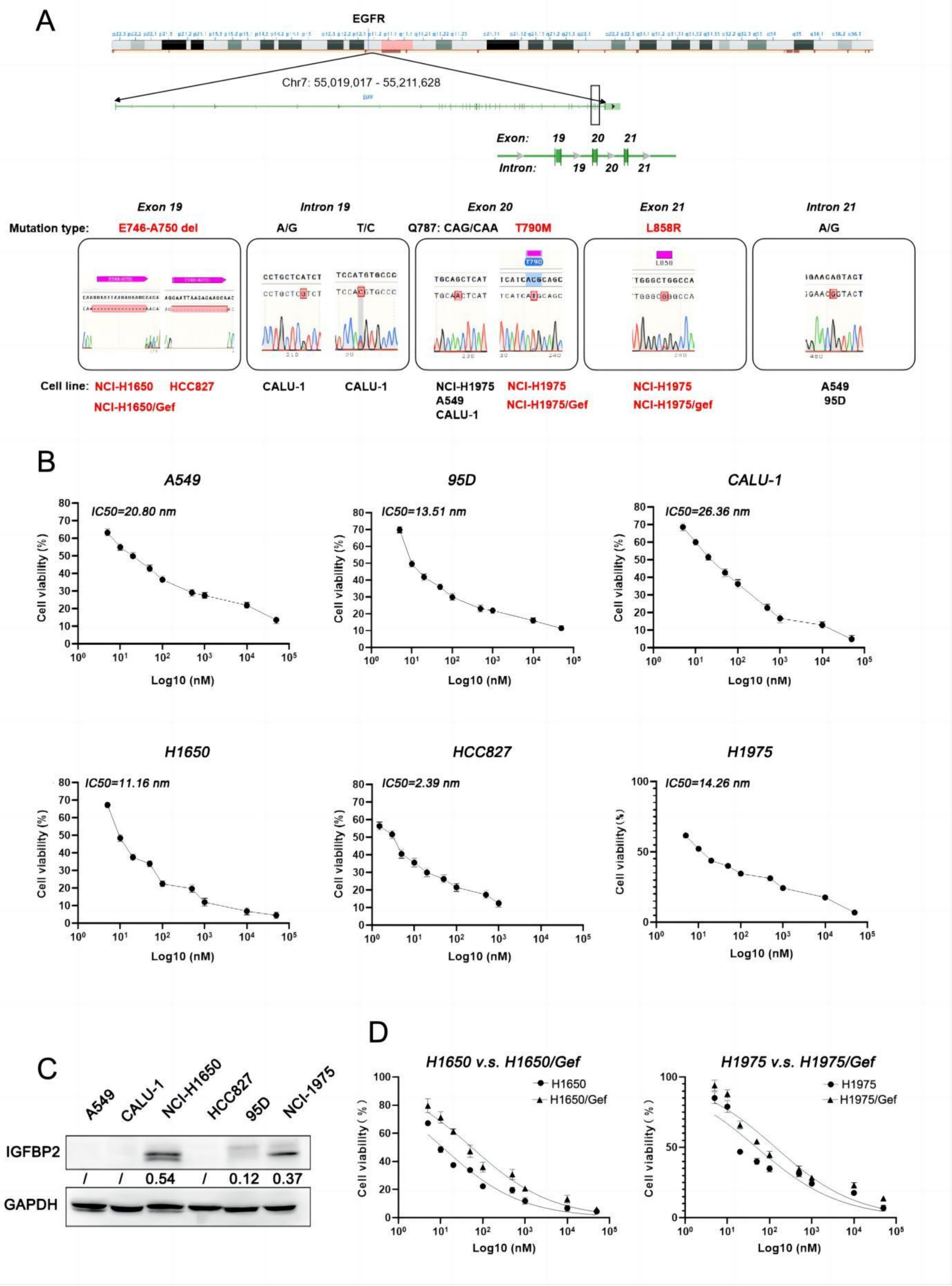

Figure S1 (A) EGFR mutations in lung cancer cell lines examined by Sanger sequencing. (B) Gefitinib IC50 values detected by CCK8 assay in each lung cancer cell line. (C) The relative protein expression of IGFBP2 in lung cancer cell lines detected by western blotting method. (D) Gefitinib RI values detected by CCK-8 assay in NCI-H1650/Gef or NCI-H1975/Gef cells.

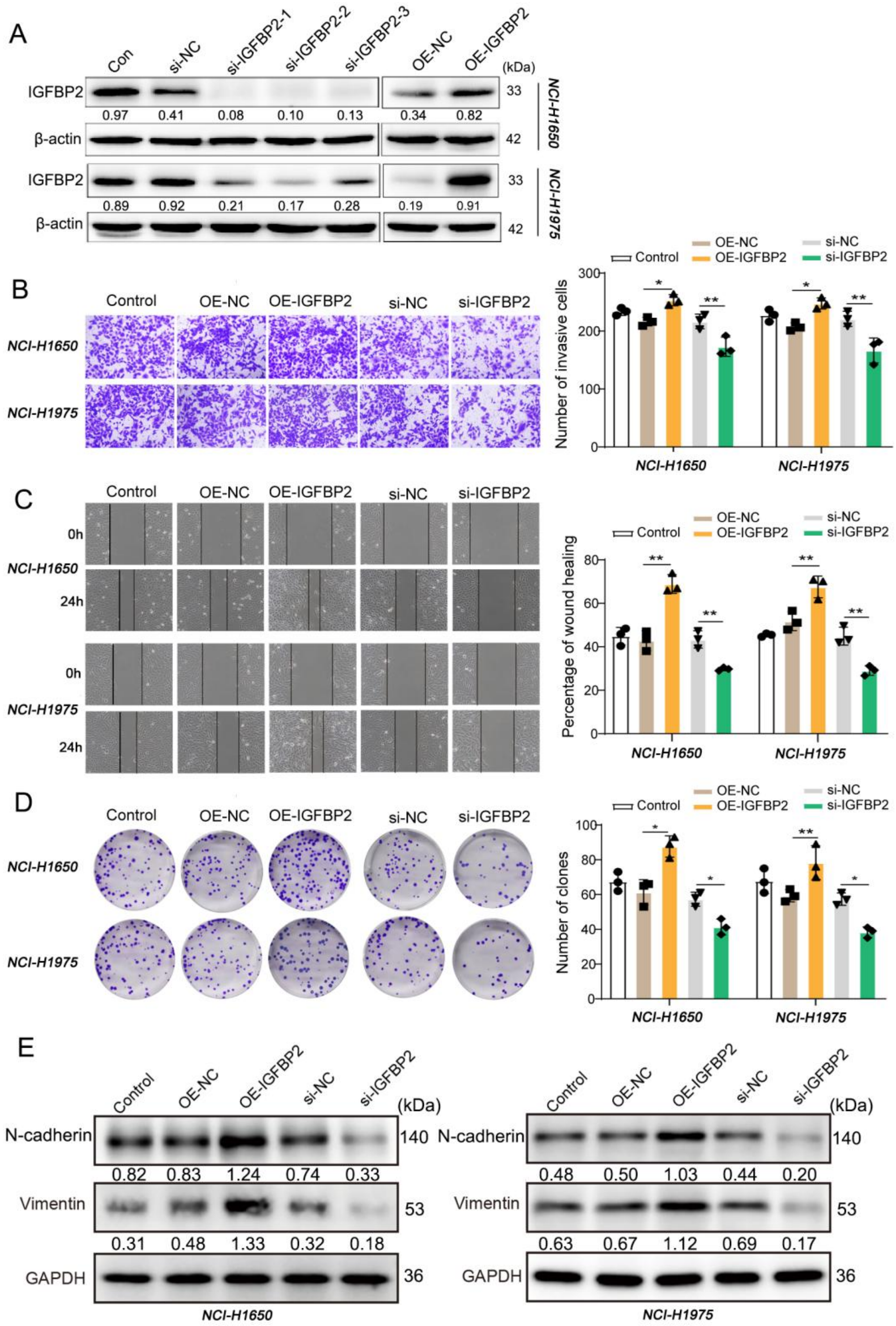

Figure S2. IGFBP2 promotes malignant phenotype of NSCLC cells. NCI-H1650 or NCI-H1975 cells were transfected with si-NC, different si-IGFBP2, overexpression vector or IGFBP2 overexpression construct. (A) The protein level of IGFBP2 was detected by western blot. (B) Cell invasion was detected by Transwell assay. (C) Cell migration was assessed by wound healing assay. (D) Colony formation was detected by colony formation assay. (D) The protein levels of N-cadherin and vimentin were detected by western blot. Dunnett's test of one-way ANOVA. *, P<0.05; **, P<0.01. OE-NC, overexpression vector alone; si-NC, negative control siRNA.

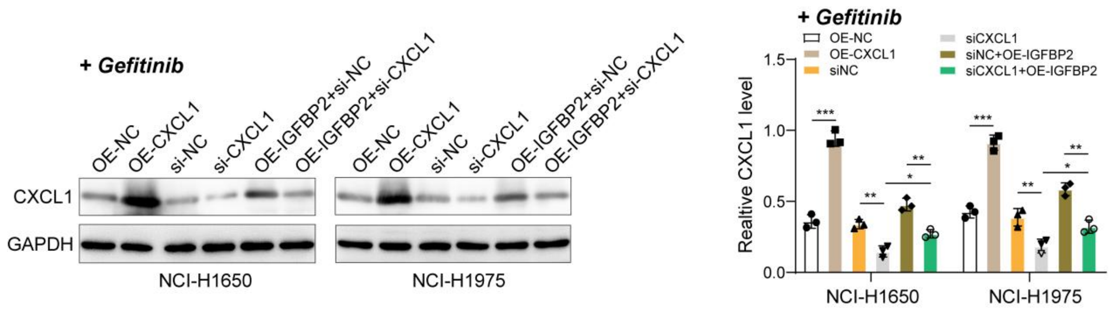

Figure S3 Validation of CXCL1 overexpression and knockdown. NCI-H1650 and NCI-H1975 cells were transfected with CXCL1 overexpression construct, si-CXCL1 or/and IGFBP2 overexpression construct. The protein level of CXCL1 was detected by western blot. Dunnett's test of one-way ANOVA. *, P<0.05; **, P<0.01; ***, P<0.001.

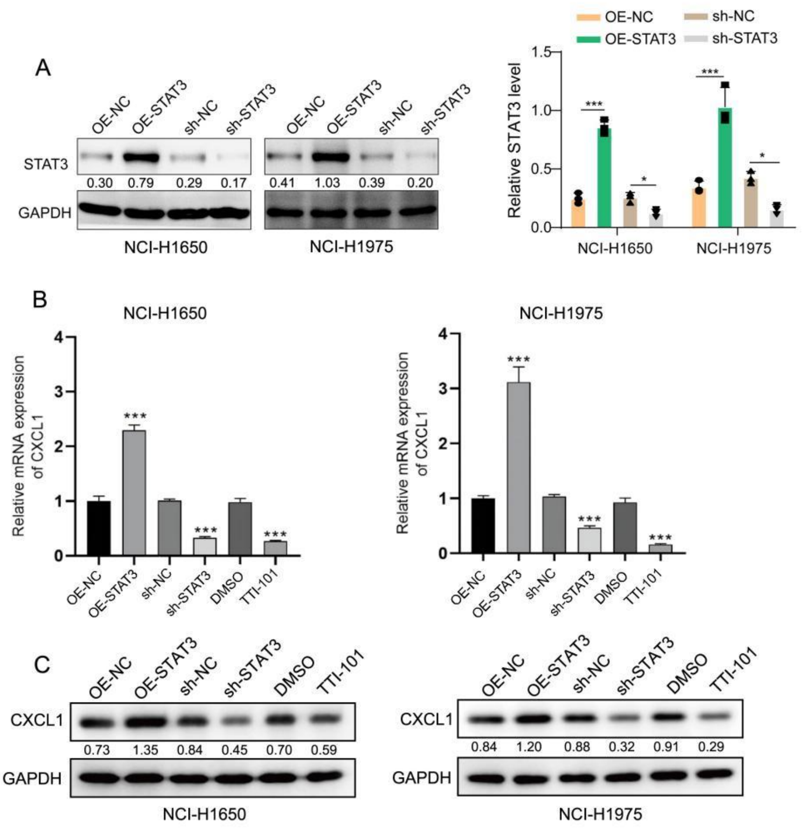

Figure S4 (A) NCI-H1650 and NCI-H1975 cells were transfected with STAT3 overexpression construct or sh-STAT3. The protein level of STAT3 was detected by western blot. STAT3 was overexpressed or silenced, or its activity was inhibited by TTI-101, and the mRNA and protein expression levels of CXCL1 were detected by QPCR (B) and Western blot (C), respectively. Dunnett's test of one-way ANOVA. *, P<0.05; **, P<0.01; ***, P<0.001.

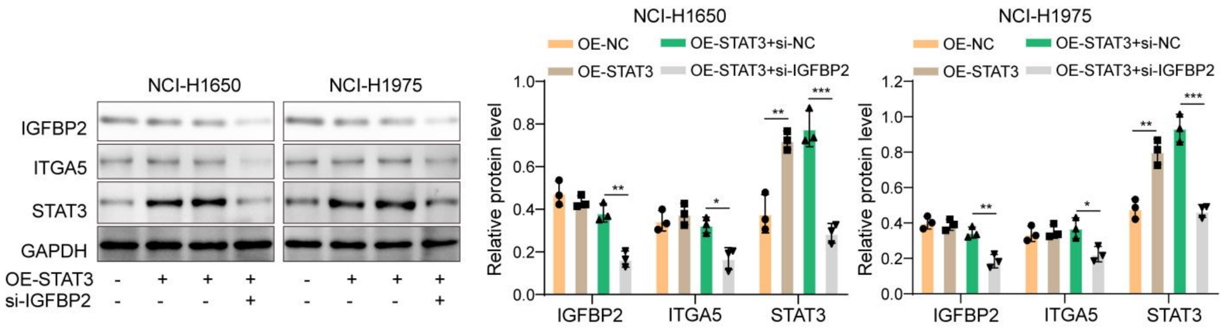

Figure S5 Effects of STAT3 overexpression or/and IGFBP2 knockdown on CXCL1 expression. NCI-H1650 and NCI-H1975 cells were transfected with STAT3 overexpression construct or si-IGFBP2. The protein levels of IGFPB2, ITGA5 and STAT3 were detected by western blot. Dunnett's test of one-way ANOVA. *, P<0.05; **, P<0.01; ***, P<0.001.

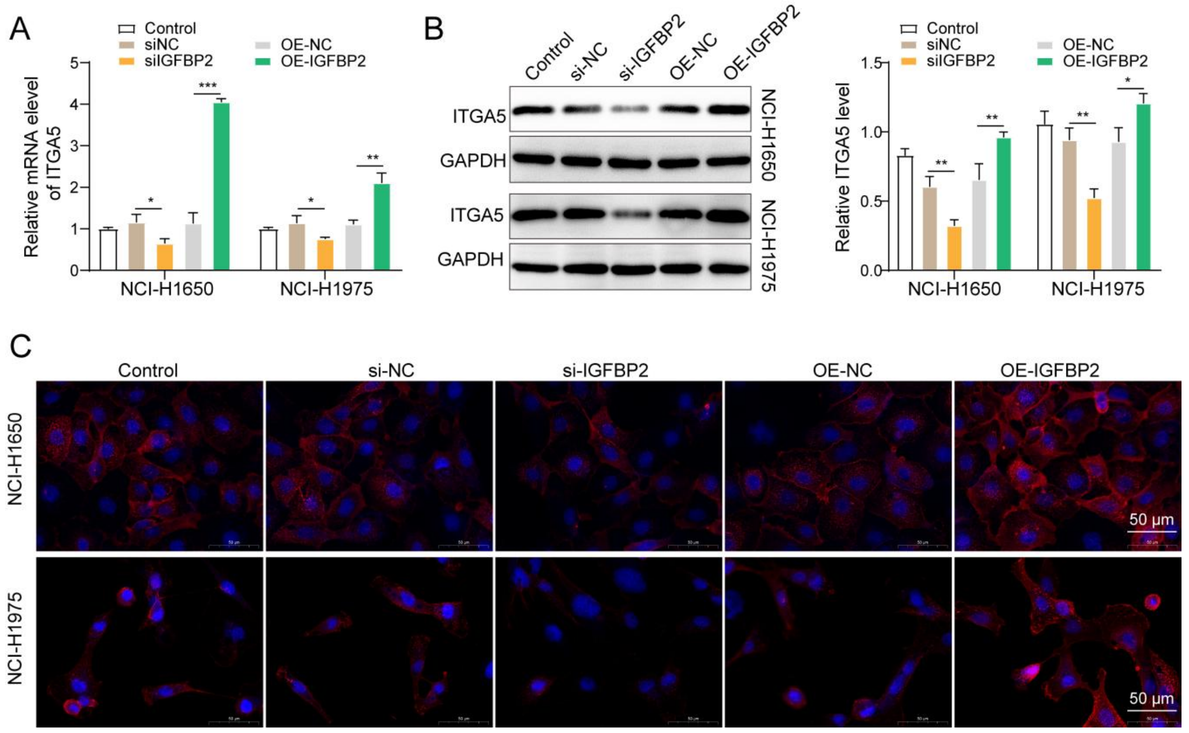

Figure S6 IGFBP2 positively regulated ITGA5 in NSCLC cells. NCI-H1650 and NCI-H1975 cells were transfected with IGFBP2 overexpression construct or si-IGFBP2. (A) The mRNA level of ITGA5 was detected by qRT-PCR. (B) The protein level of ITGA5 was detected by western blot. (C) The immunoreactivity of ITGA5 was detected by IF. Scale bar, 50 μ m . Dunnett's test of one-way ANOVA. *, P<0.05; **, P<0.01; ***, P<0.001.

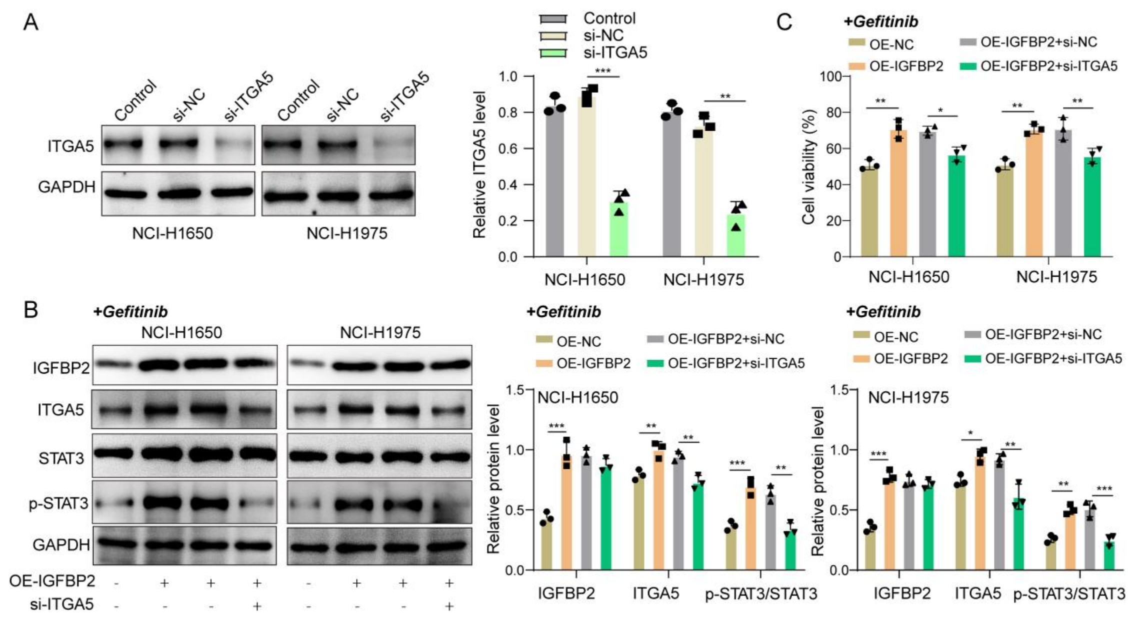

Figure S7 Effects of IGFBP2/ITGA5 axis. NCI-H1650 and NCI-H1975 cells were transfected with si-ITGA5. (A) The protein level of ITGA5 was detected by western blot. NCI-H1650 and NCI-H1975 cells were transfected with IGFBP2 overexpression construct or si-ITGA5. (B) The protein levels of IGFBP2, ITGA5, STAT3 and p-STAT3 were detected by western blot. (C) Cell viability was detected by CCK-8 assay. Dunnett's test of one-way ANOVA. *, P<0.05; **, P<0.01; ***, P<0.001.

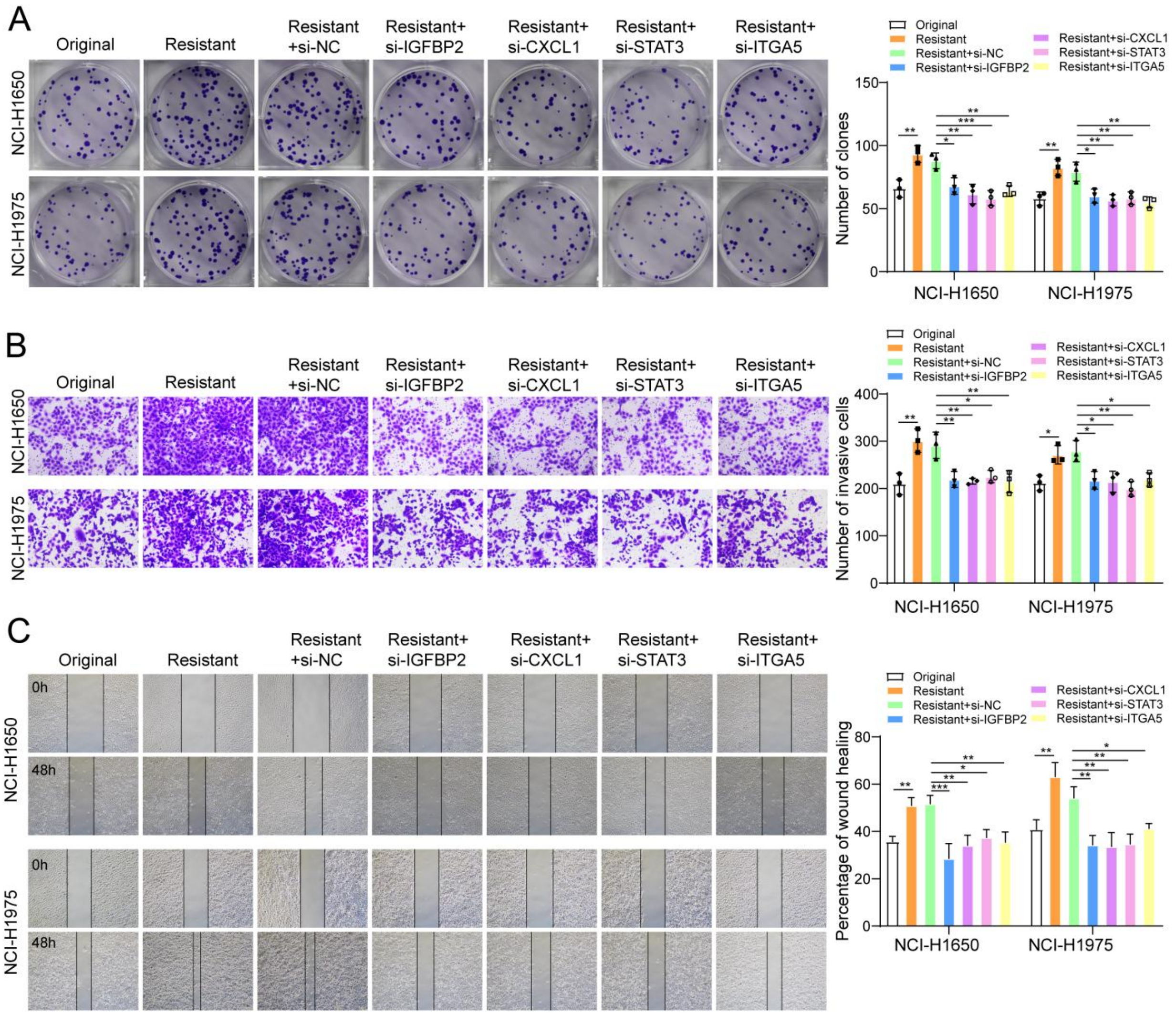

Figure.S8 NCI-H1650/Gef or NCI-H1975/Gef cells were transfected with si-NC, siRNA against IGFBP2, CXCL1, STAT3 or ITGA5. (A) Colony formation was assessed by colony formation assay with quantitative analysis. (B-C) Cell invasion and migration were detected by Transwell and wound healing assays with quantitative analysis, respectively. Dunnett's test of one-way ANOVA. *, P<0.05; **, P<0.01; ***, P<0.001. Gef, gefitinib; IC50, half maximal inhibitory concentration; si-NC, negative control siRNA.
