# Supplementary material for: IGFBP2/ITGA5 promotes gefitinib resistance via activating STAT3/CXCL1 axis in non-small cell lung cancer
Source: Cell Death Dis. 2024 Jun 25;15(6):447. doi: 10.1038/s41419-024-06843-y (PMC11199710; doi:10.1038/s41419-024-06843-y)
Supplement: Supplementary file 2 — Original Data [file 41419_2024_6843_MOESM2_ESM.pdf]

Original files of western blotting

Fig 1G

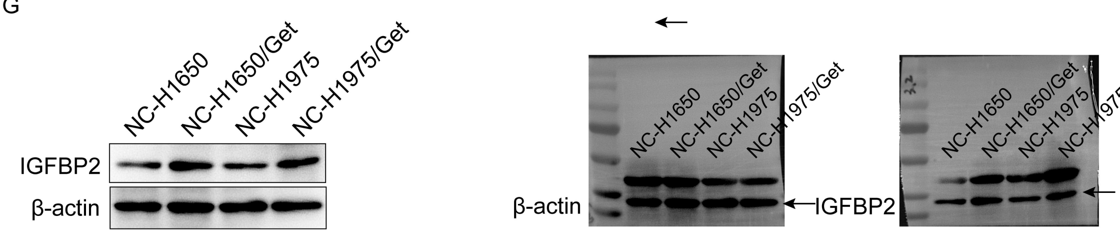

Original files of western blotting in Fig.1

Fig2D

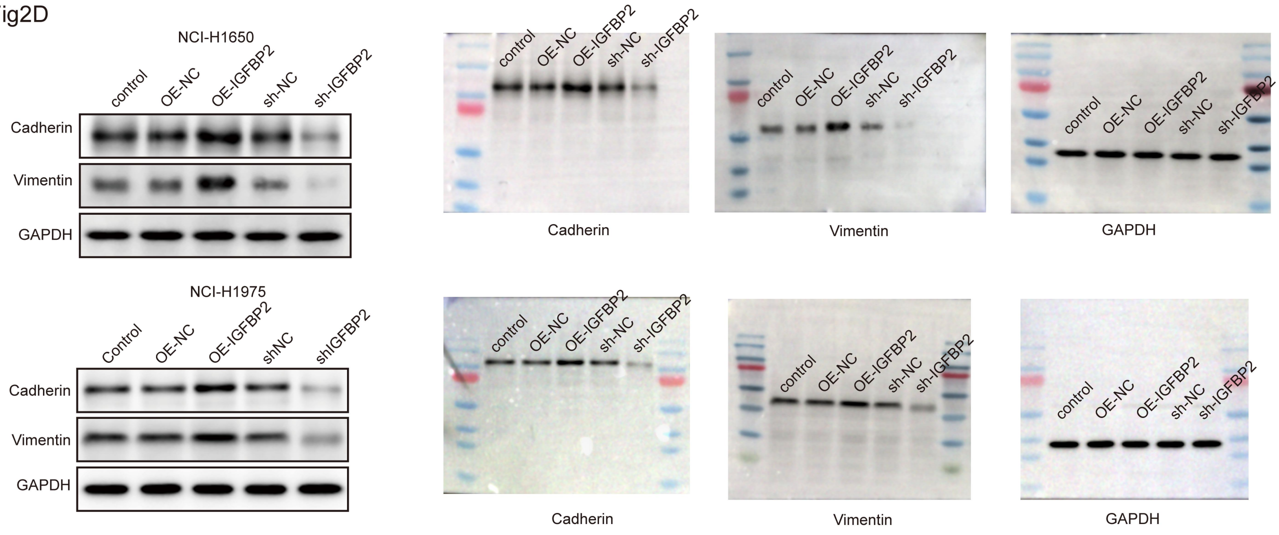

Original files of western blotting in Fig.2

Fig 3G

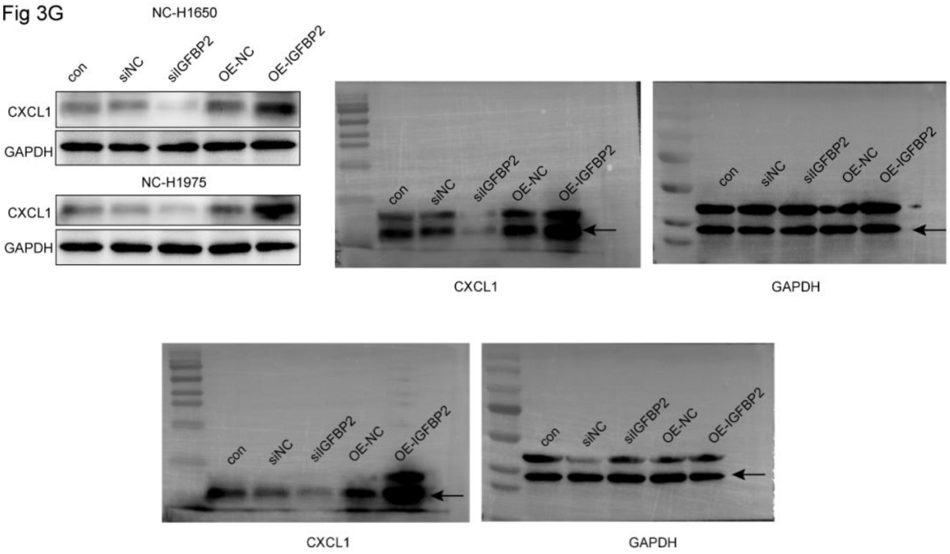

Original files of western blotting in Fig.3

Fig 4C

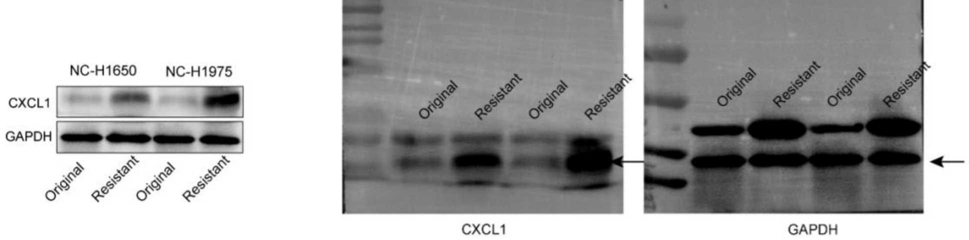

Original files of western blotting in Fig.4

Fig 5B

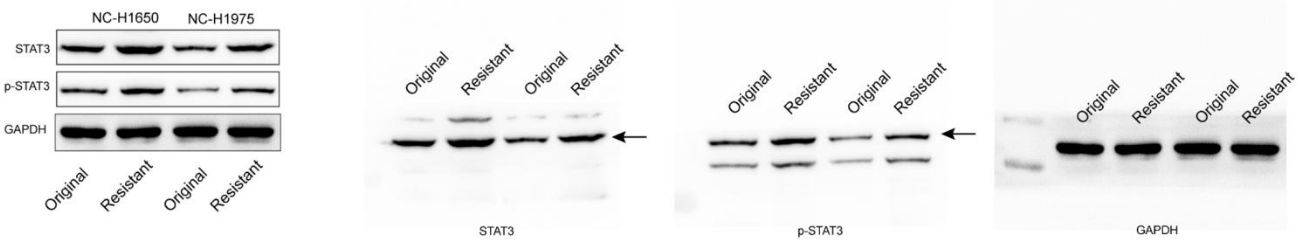

Fig 5H

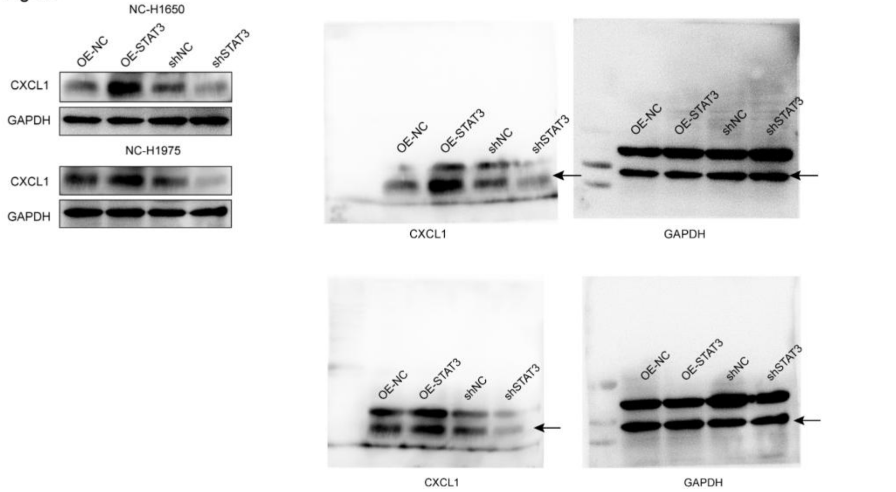

Original files of western blotting in Fig.5

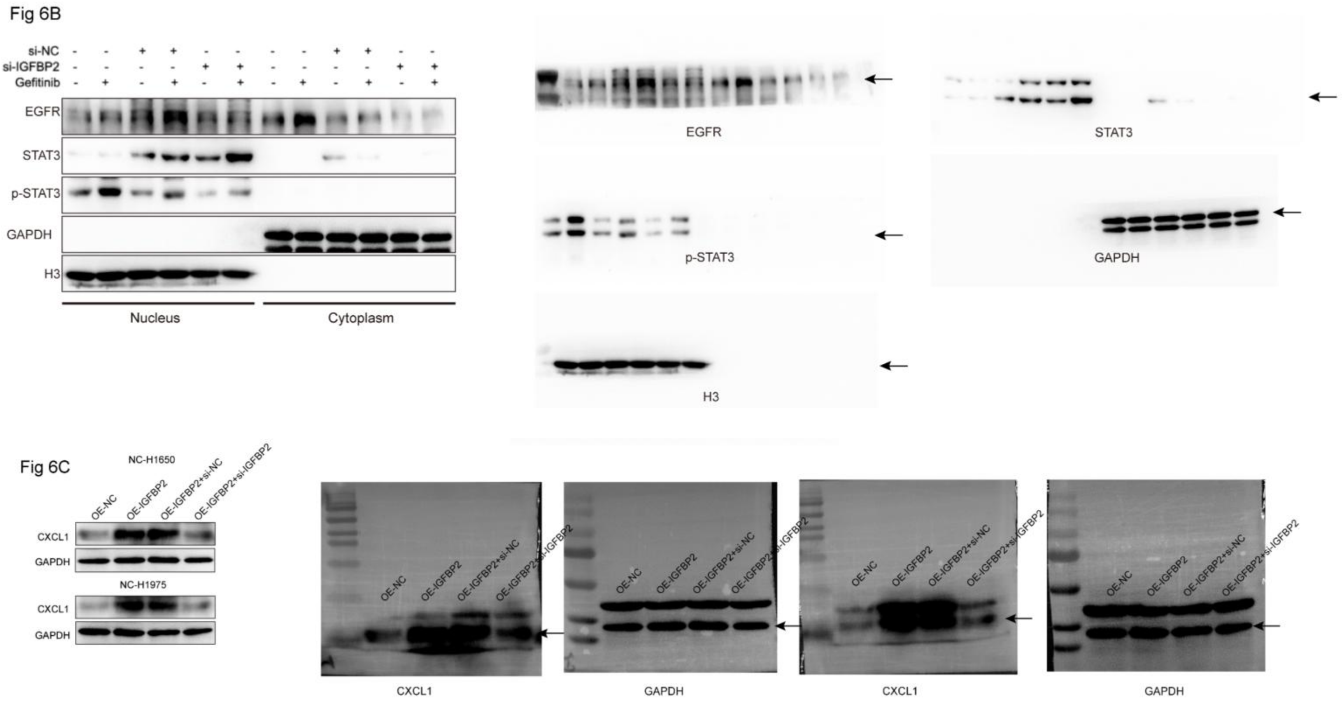

Original files of western blotting in Fig.6

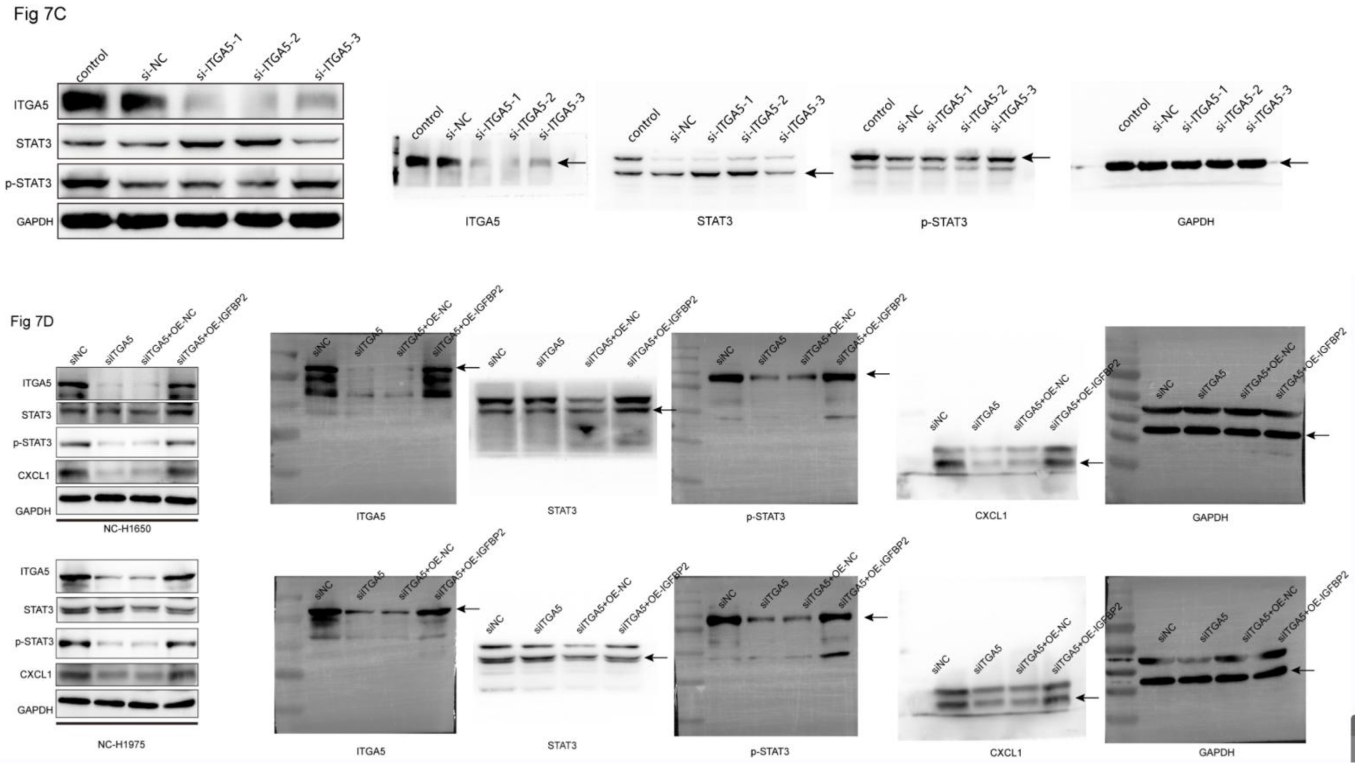

Original files of western blotting in Fig.7

fig5D

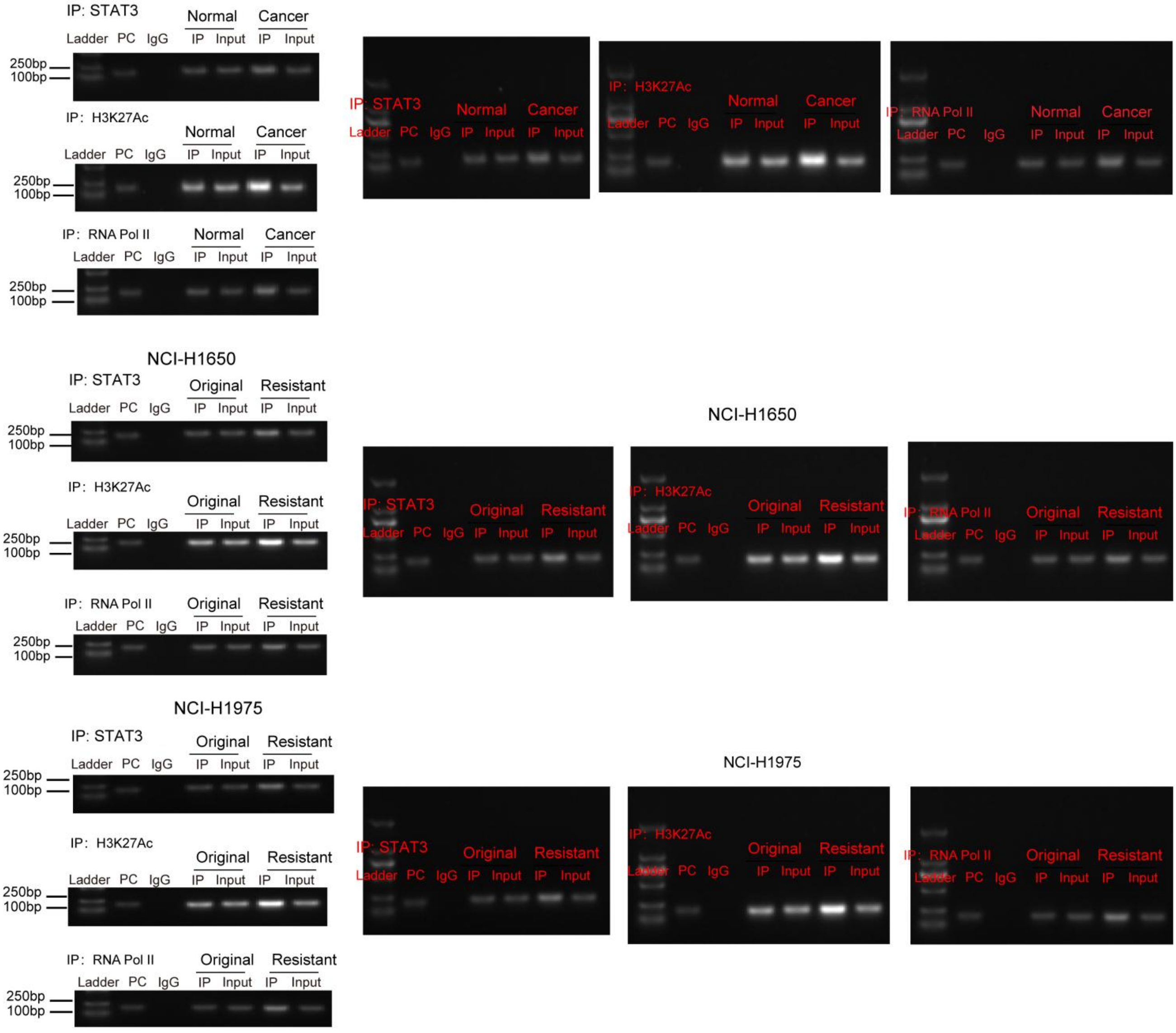

fig6A

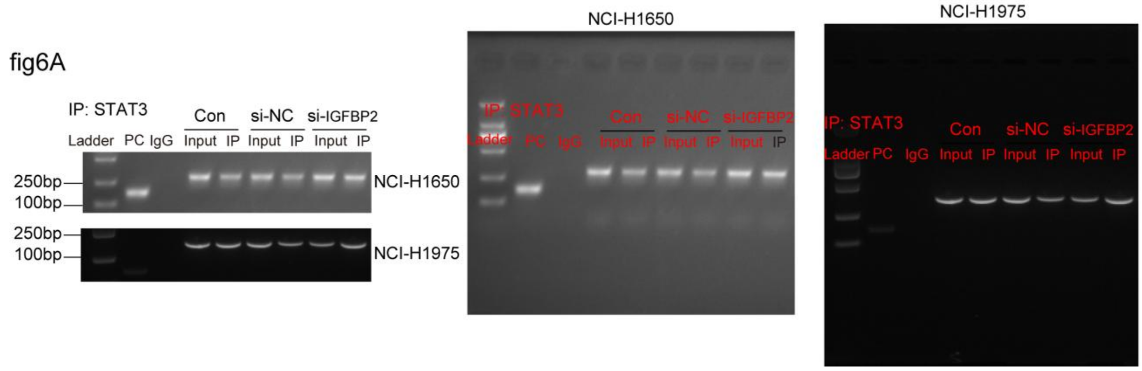

Original files of agarose gel electrophoresis images in Fig.5D and Fig.6A

Fig 8D

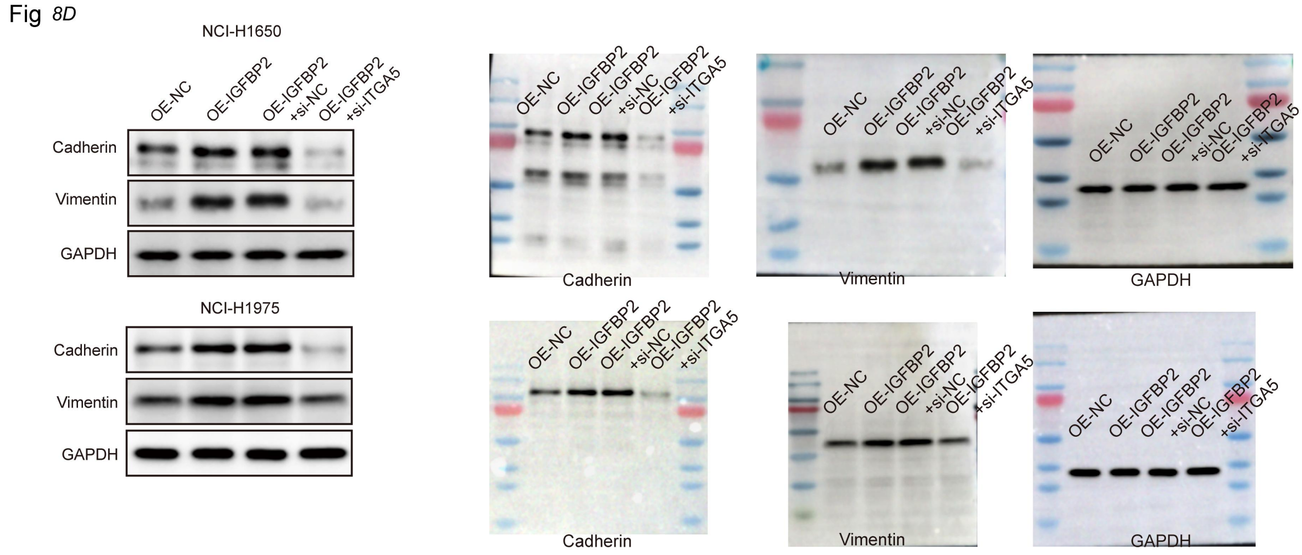

Original files of western blotting in Fig.8

Fig 9B

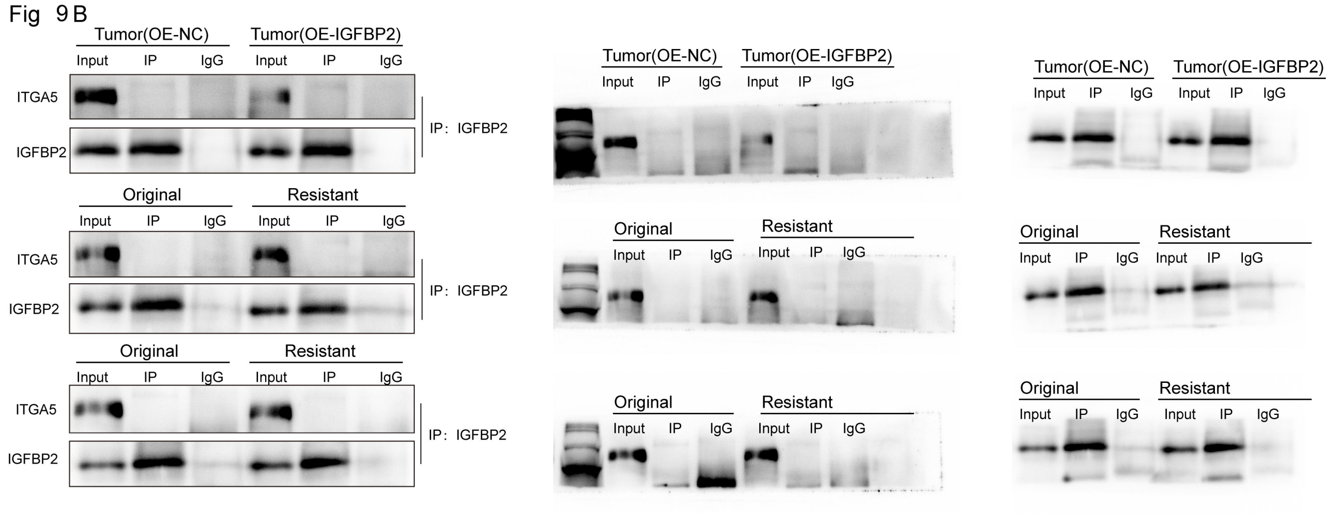

Fig 9C

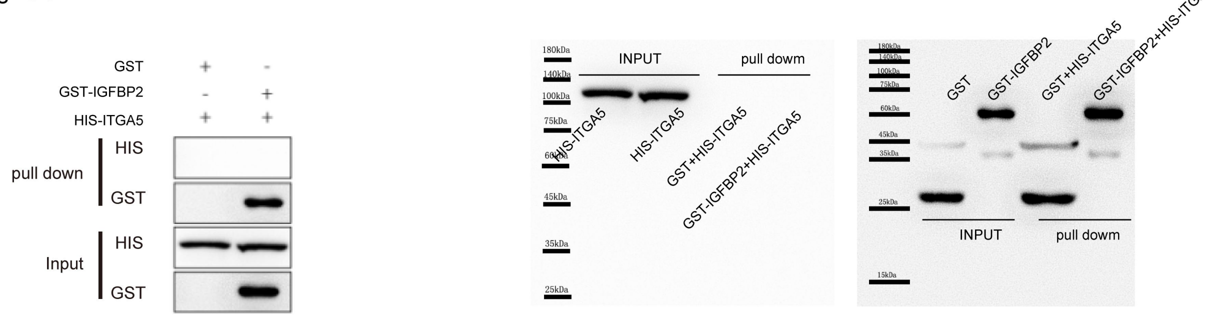

Original files of western blotting in Fig.9

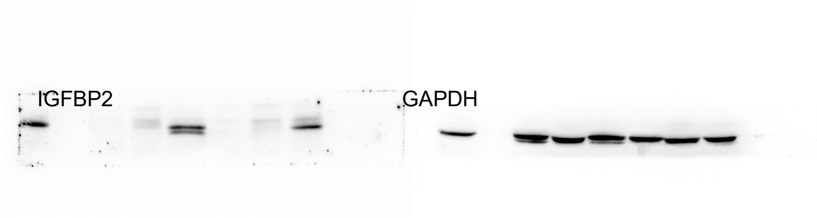

Fig S1C

Original files of western blotting in Fig.S1

Fig S2A

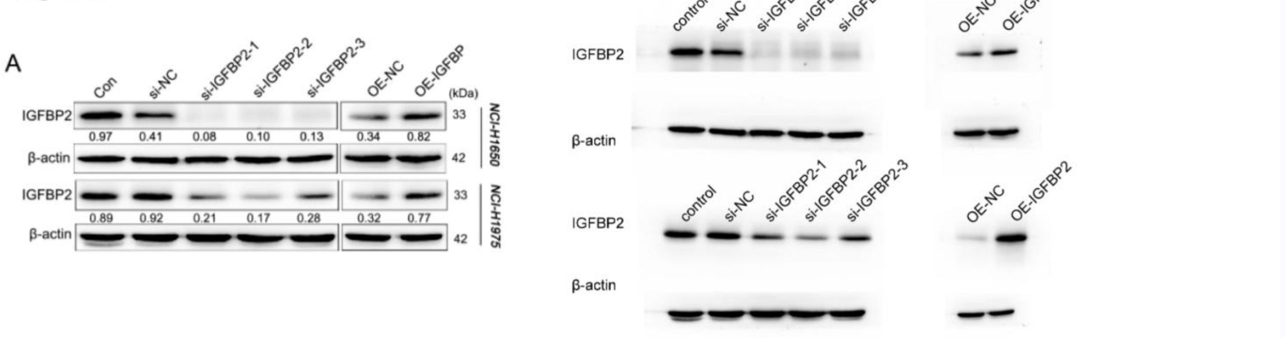

Fig S2E

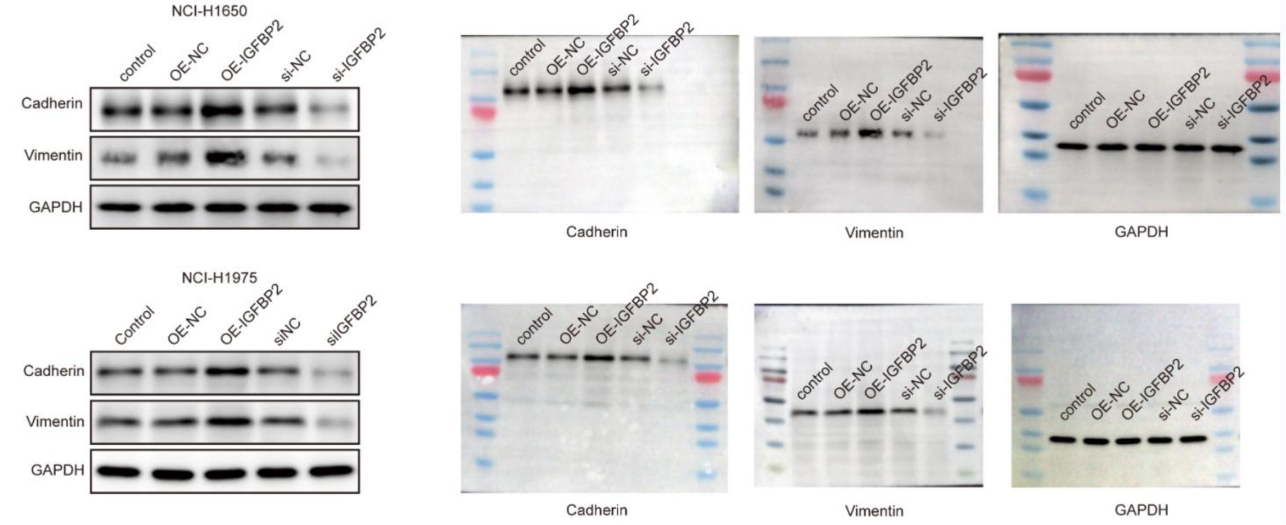

Original files of western blotting in Fig.S2

Fig S3

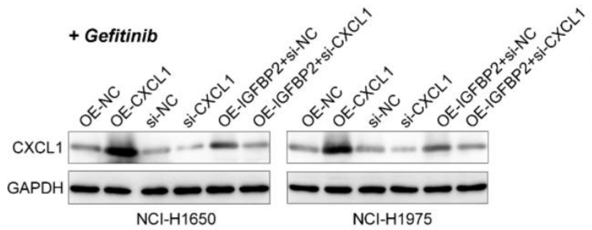

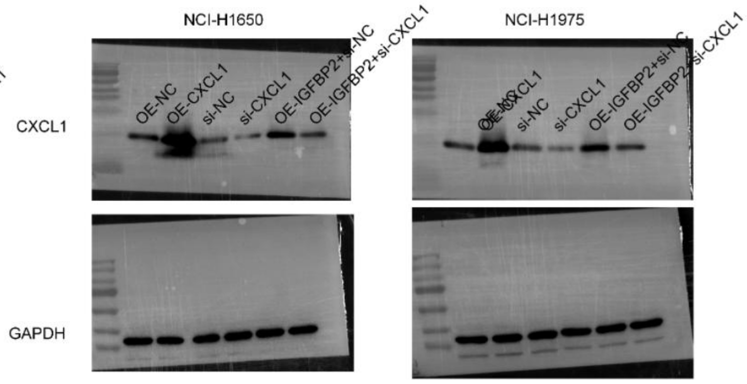

Original files of western blotting in Fig.S3

Fig S4

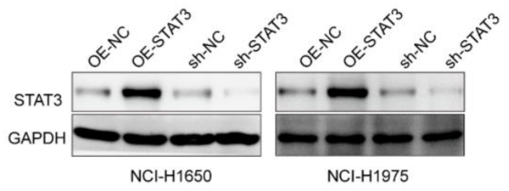

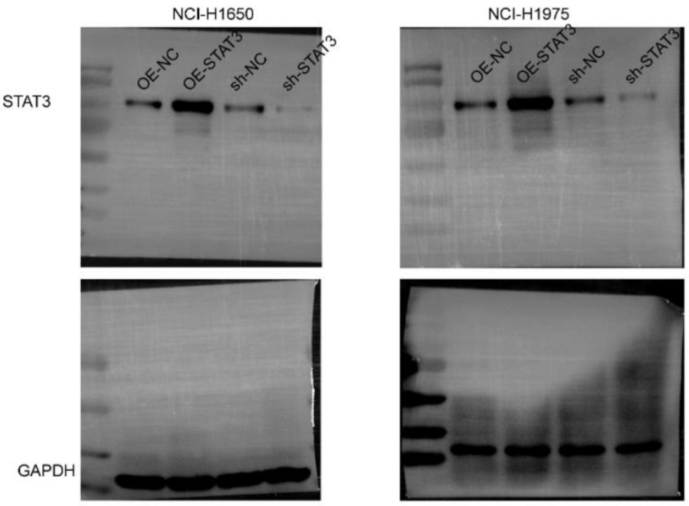

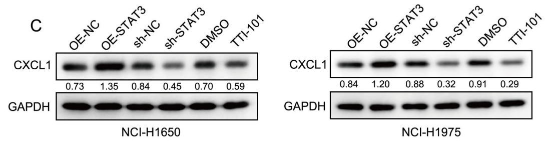

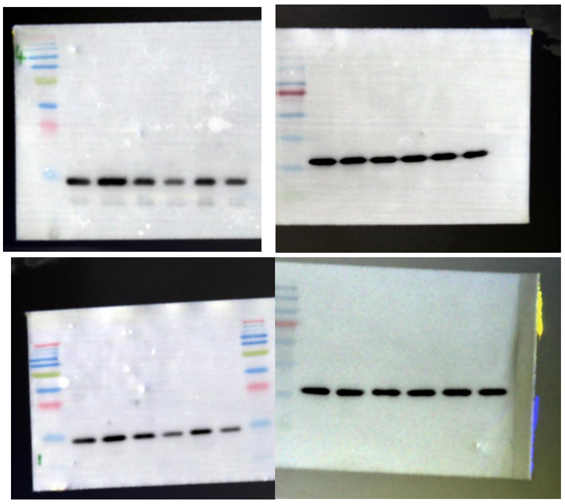

Original files of western blotting in Fig.S4

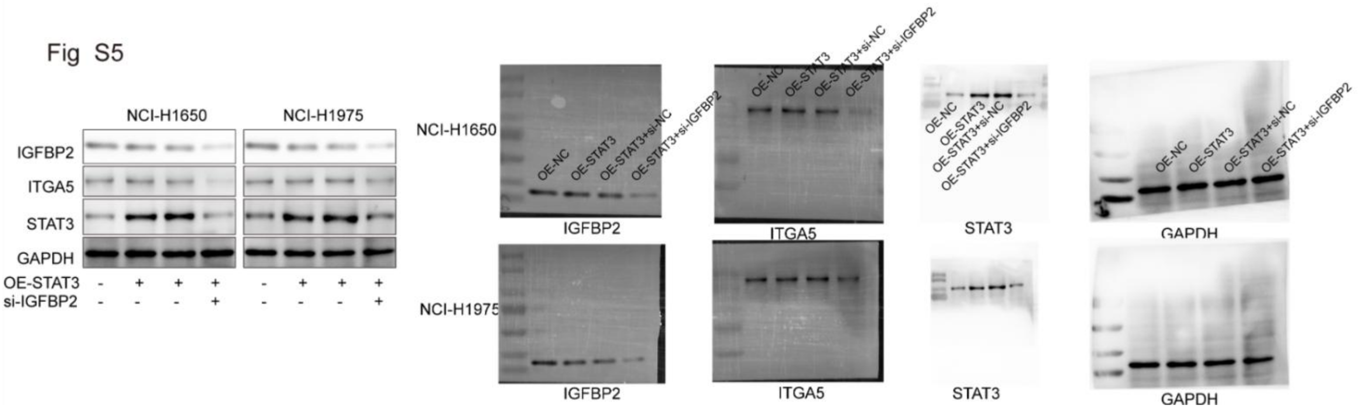

Original files of western blotting in Fig.S5

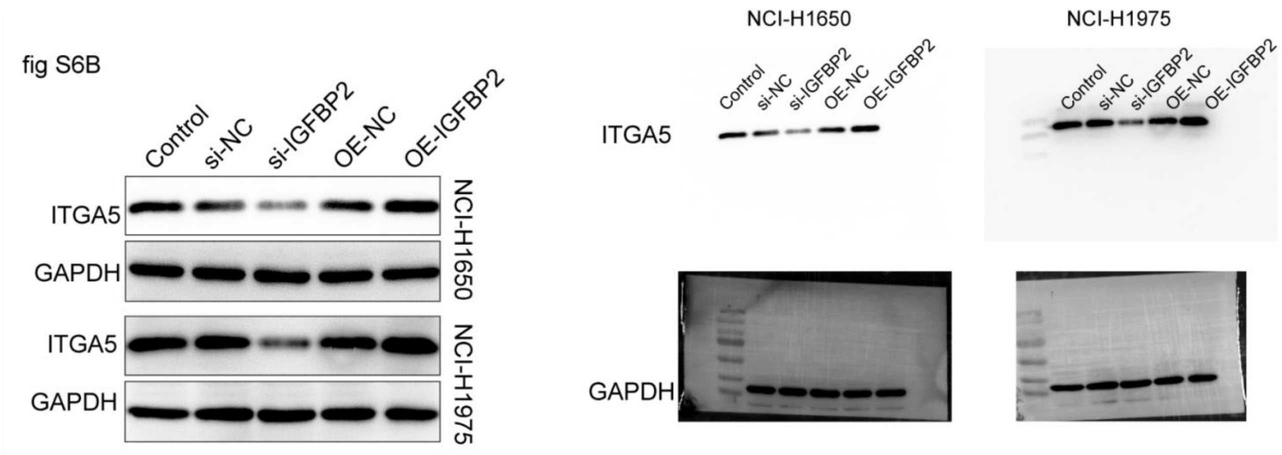

Original files of western blotting in Fig.S6

fig S7A

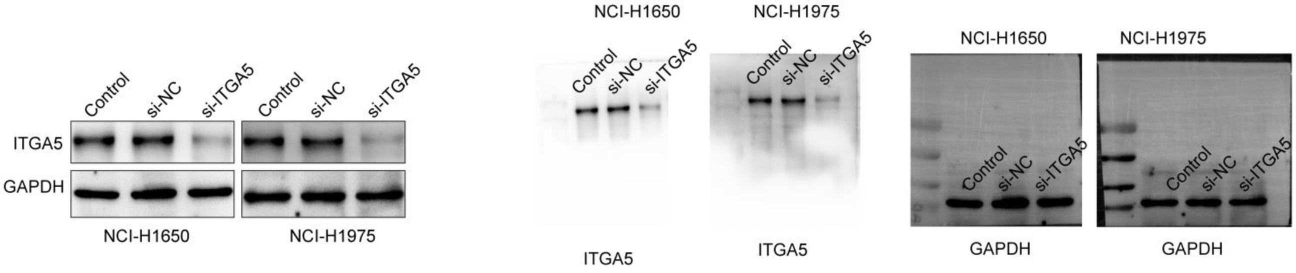

fig S7B

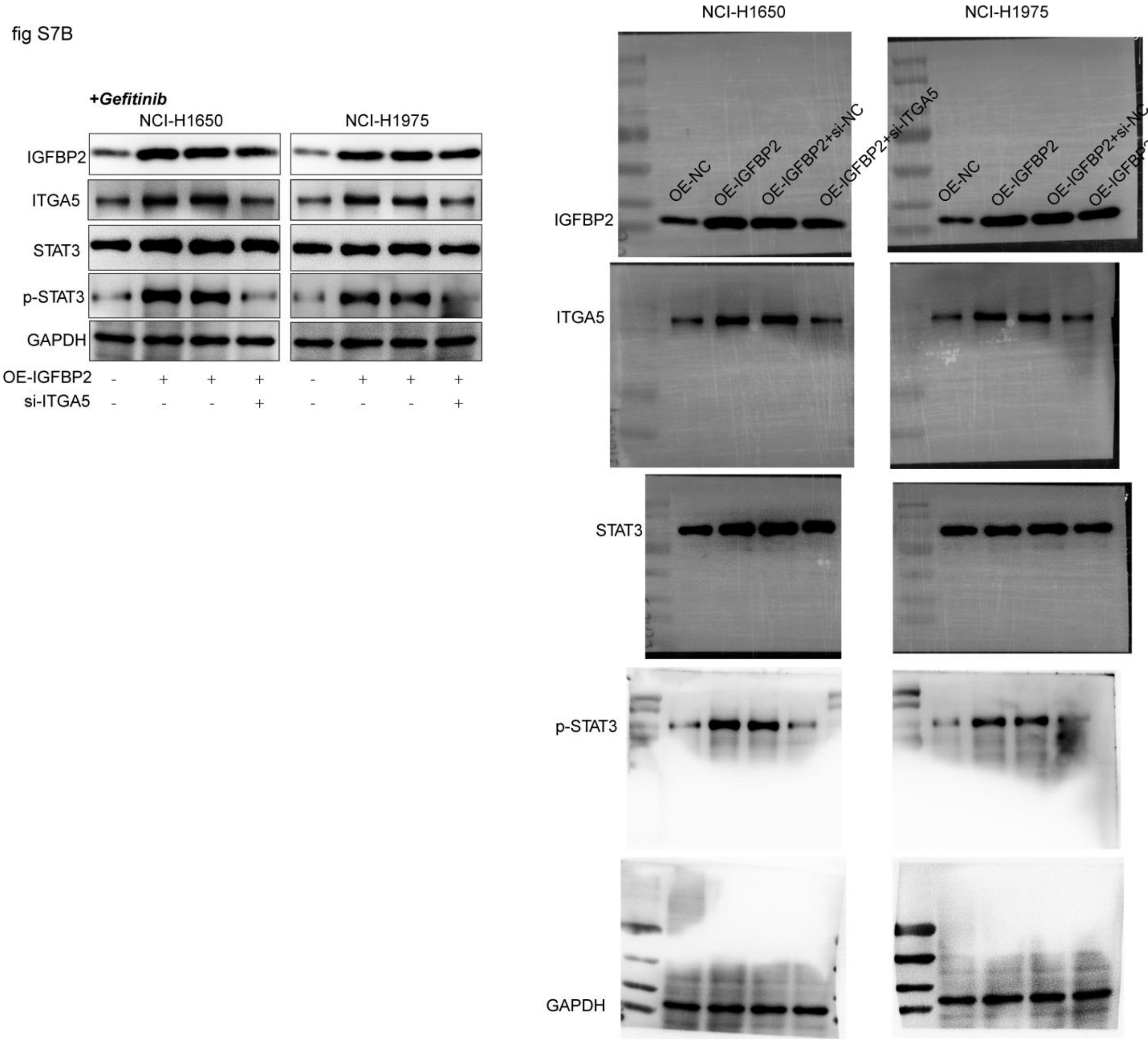

Original files of western blotting in Fig.S7
